# Uniparental expression of ribosomal RNA in ×*Festulolium* grasses: a link between the genome and nucleolar dominance

**DOI:** 10.3389/fpls.2023.1276252

**Published:** 2023-09-18

**Authors:** Václav Mahelka, David Kopecký, Joanna Majka, Karol Krak

**Affiliations:** ^1^ Czech Academy of Sciences, Institute of Botany, Průhonice, Czechia; ^2^ Institute of Experimental Botany of the Czech Academy of Sciences, Centre of Plant Structural and Functional Genomics, Olomouc, Czechia; ^3^ Faculty of Environmental Sciences, Czech University of Life Sciences Prague, Prague, Czechia

**Keywords:** ribosomal DNA, nucleolar dominance, genome dominance, *Festuca*, *Lolium*, internal transcribed spacer, genomic *in situ* hybridization, fluorescent *in situ* hybridization

## Abstract

Genome or genomic dominance (GD) is a phenomenon observed in hybrids when one parental genome becomes dominant over the other. It is manifested by the replacement of chromatin of the submissive genome by that of the dominant genome and by biased gene expression. Nucleolar dominance (ND) – the functional expression of only one parental set of ribosomal genes in hybrids – is another example of an intragenomic competitive process which, however, concerns ribosomal DNA only. Although GD and ND are relatively well understood, the nature and extent of their potential interdependence is mostly unknown. Here, we ask whether hybrids showing GD also exhibit ND and, if so, whether the dominant genome is the same. To test this, we used hybrids between *Festuca* and *Lolium* grasses (Festulolium), and between two *Festuca* species in which GD has been observed (with *Lolium* as the dominant genome in Festulolium and *F. pratensis* in interspecific *Festuca* hybrids). Using amplicon sequencing of ITS1 and ITS2 of the 45S ribosomal DNA (rDNA) cluster and molecular cytogenetics, we studied the organization and expression of rDNA in leaf tissue in five hybrid combinations, four generations and 31 genotypes [*F. pratensis* × *L. multiflorum* (F_1_, F_2_, F_3_, BC_1_), *L. multiflorum* × *F. pratensis* (F_1_), *L. multiflorum* × *F. glaucescens* (F_2_), *L. perenne* × *F. pratensis* (F_1_), *F. glaucescens × F. pratensis* (F_1_)]. We have found that instant ND occurs in Festulolium, where expression of *Lolium*-type rDNA reached nearly 100% in all F_1_ hybrids and was maintained through subsequent generations. Therefore, ND and GD in Festulolium are manifested by the same dominant genome (*Lolium*). We also confirmed the concordance between GD and ND in an interspecific cross between two *Festuca* species.

## Introduction

Interspecific hybridization is an important evolutionary phenomenon in plants. The merger of two divergent genomes into a single genetic entity, frequently accompanied by whole-genome duplication, generates genetic novelty providing raw material for selection and evolution (Gross and Rieseberg, 2005). On the one hand, the newly generated genomic diversity provides hybrid individuals with increased adaptive potential, and on the other, the unexpected complexity of the newly arisen hybrid genome may induce genetic as well as epigenetic modifications as a response to genomic instability (McClintock, 1984; Glombik et al., 2020).

Genome dominance is a phenomenon observed in hybrids when one parental genome becomes dominant over the other. Dominance can be manifested in various ways, including the physical replacement of chromatin of the submissive genome by that of the dominant genome (Zwierzykowski et al., 2006; Chalhoub et al., 2014; Majka et al., 2023) and biased gene expression (reviewed in Bird et al., 2018). Most hybrids and allopolyploids exhibit a certain degree of genome dominance (Bird et al., 2018; Alger and Edger, 2020; reviewed in Glombik et al., 2020). One example of genome dominance in allopolyploids involving both of the aforesaid mechanisms is observed in *×Festulolium* (hereafter Festulolium) – a hybrid genus between *Festuca* (fescues) and *Lolium* (ryegrasses). In Festulolium, homeologous chromosomes can pair and recombine (Kopecký et al., 2005; Kopecký et al., 2006; Kopecký et al., 2009). This opens the way for the proportion of parental chromosomes to become biased towards one parent in successive generations. It has been observed that chromosomes of *Festuca* get gradually replaced by those of *Lolium* between the F_2_ generation and the F_6_ generation, and presumably also in subsequent generations, and all commercial cultivars of *L. multiflorum × F. pratensis*, *L. multiflorum × F. glaucescens* and *L. perenne × F. pratensis* have greater numbers of *Lolium* chromosomes than *Festuca* chromosomes (Kopecký et al., 2006; Zwierzykowski et al., 2006). Another manifestation of genome dominance in Festulolium is gene expression that is biased towards the dominant *Lolium* genome; genes are more frequently expressed to the level observed in the *Lolium* parent than to the level seen in *Festuca*. This expression-level dominance of the *Lolium* genome in Festulolium hybrids occurs irrespective of chromatin elimination because it is manifested already in F_1_ hybrids with a balanced number of parental chromosomes (Stočes et al., 2016; Glombik et al., 2021).

Another classic phenomenon observed in hybrids and allopolyploids is nucleolar dominance (ND). It concerns ribosomal DNA and entails the formation of nucleoli by nucleolar organizing regions (NORs) inherited from only one parental species of a hybrid (Navashin, 1934; Borowska-Zuchowska et al., 2023). Nucleoli are sites of ribosomal RNA gene transcription and ribosome assembly. Therefore, only functional, transcriptionally active NORs give rise to nucleoli. A typical feature of ND is that ribosomal genes inherited from one (dominant) parental species are expressed, while those inherited from the other are silenced (Volkov et al., 2007). The mechanisms responsible for the silencing of rRNA genes are well understood and involve increased chromatin condensation, histone deacetylation and DNA methylation (reviewed in Grummt and Pikaard, 2003; Pikaard, 2018). However, the central question remains largely unanswered: What is the mechanism controlling which gene subset should be expressed and which should be silenced? It has been suggested that the intergenic spacer (IGS) region, in which the spacer and the gene promoters are located, plays a central role in rDNA transcription through interactions of sequence and repeats with transcription factors, or through the production of small interfering RNAs that mediate DNA methylation (Pikaard, 2000; Tucker et al., 2010). However, other studies documented caveats in the particular hypotheses (e.g. Chen et al., 1998; Chandrasekhara et al., 2016), suggesting that neither of the hypotheses is generally applicable. In particular, evidence from studies on *Arabidopsis* indicates that ND is enforced independently of both transcription factor availability and binding affinity (Chen et al., 1998). In F_1_ hybrids, silencing of alleles from the submissive genome is highly variable, with two generations needed to establish ND in some lines. After backcrossing to the submissive parent, the direction of ND can be reversed, clearly demonstrating a gene or genome dosage effect. Furthermore, tissue specificity of ND may occur, as shown, for example, in *Brachypodium hybridum*, where ND was shown to be stable in leaves but not in roots (Borowska-Zuchowska et al., 2021). Tissue specificity of ND seems to be commonplace in hybrids displaying ND, including *Arabidopsis* (Pontes et al., 2007), *Brassica* (Hasterok and Maluszynska, 2000a), *Urochloa* (Santos et al., 2020), *Solanum* (Komarova et al., 2004), *Allium* (Hasterok and Maluszynska, 2000b), *Tragopogon* (Dobešová et al., 2015) and *Brachypodium* (Borowska-Zuchowska et al., 2021).

Obviously, ND is a complex phenomenon in which control on a larger, chromosomal scale takes place. Possibly, the effect of the chromosomal position of rDNA sites is critical (Mohannath et al., 2016; Báez et al., 2020; Saradadevi et al., 2020), indicating that rather than rDNA sequences themselves, DNA sequences spanning the rDNA array may determine the activity of the NOR (Chandrasekhara et al., 2016). The hypothesis suggesting that NOR inactivation stems from the spread of silencing from adjacent chromosomal regions was undermined in a study documenting the expression of protein-coding genes located in a 3.1 kb proximity from rRNA gene sequences that were silenced (Lewis and Pikaard, 2001). From an extreme point of view, the activity of the NOR might be affected by genome-wide changes associated with interspecific hybridization. It has been demonstrated that following a genome merger, one of the parental genomes (the submissive one) displays increased histone methylation and its chromatin becomes more compact (Zhu et al., 2017). Apparently, the observed feature was enforced by factors of the dominant (up-regulated) genome, suggesting that the ND was governed by the same mechanisms as genome-wide dominance. Thus, the question arises: Do species manifesting genome dominance also display ND, and if so, is the dominant genome the same? Both the presence and stability of *Lolium*-genome dominance in Festulolium (Glombik et al., 2020; Glombik et al., 2021; Majka et al., 2023) make it a suitable model for studying the potential relationship between nucleolar and genomic dominance. Therefore, in this study, we use synthetic hybrids involving both fescues (namely *Festuca pratensis* and *F. glaucescens*) and ryegrasses (*Lolium multiflorum* and *L. perenne*) to study the organization and expression of ribosomal DNA (rDNA) in different hybrid combinations and generations. To fulfil the goal, we used a combination of sequencing (Illumina amplicon sequencing of the internal transcribed spacers ITS1 and ITS2 of 45S rDNA) and molecular cytogenetics (genomic *in situ* hybridization and fluorescent *in situ* hybridization with a 45S rDNA probe) to explore the following questions: (1) What is the organization and sequence variation of 45S rDNA in parental taxa and their hybrids? (2) What is the expression pattern of 45S rDNA in different hybrid combinations? (3) In hybrids for which series of successive and backcross hybrid generations are available, is the observed pattern of rDNA expression stable in particular generations?

## Materials and methods

### Plant material selection and cultivation

In this study, we used multiple genotypes of hybrids between *Festuca* and *Lolium*, and between two *Festuca* species. The hybrids come from the collection of D. Kopecký (IEB, Olomouc), produced for the purpose of studying genome dominance in Festulolium (e.g., Glombik et al., 2021; Majka et al., 2023). The core hybrid combination was that of *F. pratensis* × *L. multiflorum*, for which F_1_, F_2_, F_3_, and backcross BC_1_ (F_1_ hybrid ♀ × *L. multiflorum* ♂) genotypes were available. Apart from this one, the following hybrid combinations were used (particular generations used for each combination are given in brackets): *L. multiflorum* × *F. pratensis* (F_1_), *L. multiflorum* × *F. glaucescens* (F_2_), *L. perenne* × *F. pratensis* (F_1_) and *F. pratensis* × *F. glaucescens* (F_1_). All of the genotypes are tetraploids (*F. glaucescens* is segmental allotetraploid, Kopecký et al., 2009), except diploid hybrids *Lolium perenne* × *Festuca pratensis*. Although the dominance of the *Lolium* genome over the *Festuca* genome at the chromosome level has already been observed (reviewed in Majka et al., 2020), we also incorporated a hybrid of two *Festuca* species (*F. pratensis* and *F. glaucescens*, both submissive in *Festuca* × *Lolium* hybrids) in this study to take a broader view of the level and interdependence of genomic and nucleolar dominance. The plants used in this study were 2–3 years old. For each cross and generation, we used between one and six genotypes in order to account for possible variation among different individuals. The full list of plant material, including the origin of particular genotypes, is given in **Table 1**.

**Table 1:** Characteristics of the Festulolium genotypes used in this study.

cross, parent	genotype	generation	genotype origin
*F. pratensis* (4x) *× L. multiflorum* (4x)	FL_06	F1	Fp WESTA (FL_39) × Lm
	FL_07	F1	Fp WESTA (FL_39) × Lm
	FL_08	F1	Fp WESTA (FL_39) × Lm
	FL_09	F1	Fp WESTA (FL_39) × Lm
	FL_10	F1	Fp WESTA (FL_39) × Lm
	FL_18	F2	selfing of F1 (FL_07)
	FL_19	F2	selfing of F1 (FL_09)
	FL_26	F3	F3 derived from FL_08
	FL_20	BC	BC_F1 × Lm
	FL_21	BC	BC_F1 × Lm
	FL_22	BC	BC_F1 × Lm
	FL_23	BC	BC_F1 × Lm
	FL_24	BC	BC_F1 × Lm
	FL_25	BC	BC_F1 (FL_06) × Lm
*L. multiflorum* (4x) *× F. pratensis* (4x)	FL_11	F1	Lm × Fp WESTA (FL_39)
	FL_12	F1	Lm × Fp WESTA (FL_39)
*L. perenne* (2x) *× F. pratensis* (2x)	FL_13	F1	Lp MATIZ × Fp WSC
	FL_14	F1	Lp MATIZ × Fp WSC
	FL_15	F1	Lp MATIZ × Fp WSC
	FL_16	F1	Lp MATIZ × Fp WSC
	FL_17	F1	Lp MATIZ × Fp WSC
*L. multiflorum* (4x) *× F. glaucescens* (4x)	FL_27	F2	selfing of F1 (Lm × Fg)
	FL_28	F2	selfing of F1 (Lm × Fg)
	FL_29	F2	selfing of F1 (Lm × Fg)
	FL_30	F2	selfing of F1 (Lm × Fg)
	FL_41	F2	selfing of F1 (Lm × Fg)
*F. glaucescens* (4x) *× F. pratensis* (4x)	FL_01	F1	Fg (FL33) × Fp WESTA
	FL_02	F1	Fg (FL33) × Fp WESTA
	FL_03	F1	Fg (FL33) × Fp WESTA
	FL_04	F1	Fg (FL33) × Fp WESTA
	FL_05	F1	Fg (FL33) × Fp WESTA
*L. multiflorum* (4x)	FL_37	Parent_LM	cv. PODIUM
	FL_42	Parent_LM	cv. PODIUM
	FL_43	Parent_LM	cv. PODIUM
*F. pratensis* (4x)	FL_34	Parent_FP	cv. WESTA
	FL_35	Parent_FP	cv. WESTA
	FL_36	Parent_FP	cv. WESTA
	FL_39	Parent_FP	cv. WESTA
*F. glaucescens* (4x)	FL_31	Parent_FG	ecotype
	FL_32	Parent_FG	ecotype
	FL_33	Parent_FG	ecotype
*L. perenne* (2x)	FL_38	Parent_LP	cv. MATIZ
*F. pratensis* (2x)	FL_40	Parent_FP	cv. WSC

Lm, *Lolium multiflorum*; Lp, *L. perenne*; Fp, *Festuca pratensis*; Fg, *F. glaucescens*. cv., cultivar.

The plants were grown in 10 × 10 cm plastic pots in a mixture of compost and sand (2:1) in the Experimental garden of the Institute of Botany, Průhonice (Czechia). Prior to DNA extraction, the pots were placed in a growth chamber and cultivated at 20/16°C (day/night), 16-h light regime for 21 days to keep the plants in unified conditions.

### Cytogenetic analyses (GISH and FISH)

Genomic and fluorescent *in situ* hybridization (GISH, FISH) experiments were carried out to determine the genomic composition of the hybrids and to investigate the number of sites with rDNA loci in parental species and hybrids. Plants were transferred to a hydroponic culture with an aerated solution of Hydroponex at 0.9 g/l (Hu-Ben, Čerčany, Czech Republic). After five to seven days, actively growing root tips were collected to ice water for 26–28 hours, fixed in a 3:1 mixture of absolute ethanol and glacial acetic acid at 37°C for seven days, stained in 1% acetocarmine for two hours and squashed in a drop of 45% acetic acid on clean microscope slides. FISH was done according to Masoudi-Nejad et al. (2002). Total genomic DNA of *F. pratensis* (*F. pratensis × L. multiflorum* and *L. perenne × F. pratensis* hybrids) or *F. glaucescens* (*F. glaucescens × F. pratensis* and *L. multiflorum × F. glaucescens* hybrids) was labelled with digoxigenin using the DIG-Nick Translation Kit according to manufacturer’s recommendation (Roche) and used as a probe. DNA clone pTa71 (Gerlach and Bedbrook, 1979) containing a 9-kb *Eco*RI fragment of wheat ribosomal DNA, which carries the 18S-5.8S-26S cluster of ribosomal RNA genes (here referred to as 45S rDNA), was labelled with biotin using the biotin-Nick Translation Kit (Roche). Genomic DNA of *L. multiflorum* (*F. pratensis × L. multiflorum, L. multiflorum × F. glaucescens*, and *L. perenne × F. pratensis* hybrids) or *F. pratensis* (*F. glaucescens × F. pratensis* hybrids) were sheared to 200–500-bp fragments by boiling for 45 min and used as blocking DNA. Sites of probe hybridization were detected by the Anti-DIG-FITC conjugate (Roche) and streptavidin-Cy3 conjugate (Amersham). Chromosomes were counterstained with 1.5-µg/ml 4′,6-diamidino-2-phenylindole (DAPI) in Vectashield antifade solution (Vector Laboratories). Slides were evaluated with an Olympus AX70 microscope equipped with epi-fluorescence and a SensiCam B/W camera. ScionImage and Adobe Photoshop software were used for the processing of color images.

### Inference of genome dominance in *L. multiflorum* × *F. glaucescens* and *F. glaucescens* × *F. pratensis* hybrids

One of the manifestations of genomic dominance in Festulolium is the physical replacement of chromatin of the submissive genome by that of the dominant genome. This has been demonstrated in a series of previous studies (see Introduction). In this study, we used GISH to determine for the first time the genomic composition of successive generations of *L. multiflorum × F. glaucescens* and *F. glaucescens × F. pratensis* hybrids. For this purpose, we compared the genomic composition of 72 *L. multiflorum × F. glaucescens* and 9 F*. glaucescens × F. pratensis* hybrids of F_1_ and F_2_ generations. GISH was done as described in the previous chapter.

### DNA and RNA extraction and cDNA synthesis

From each plant, we sampled two mature leaves, which were flash frozen in liquid nitrogen and stored at −80°C until DNA and RNA extraction. DNA and RNA extractions were done using the DNeasy Plant Mini Kit (Qiagen, Hilden, Germany) and the NucleoSpin RNA Plus Mini kit (Macherey-Nagel, Düren, Germany), respectively, following the protocols provided by the manufacturers. Contaminating genomic DNA was removed from the RNA samples by treatment with the TURBO DNA-Free Kit (Thermo Fisher Scientific, Waltham, MA, USA), and cDNA was then synthesized using the Transcriptor High Fidelity cDNA Synthesis Kit (Roche, Basel, Switzerland) based on random hexamer priming according to the manufacturer’s instructions.

### rDNA amplification and sequencing

To investigate ND in Festulolium, we analyzed ITS1 and ITS2 regions of 45S rDNA. Both regions were amplified separately using genomic DNA (gDNA) and complementary DNA (cDNA) as templates. ITS1 was amplified using the grass-specific primer ITS_PoaF (Mahelka et al., 2007, 5′ AAGGATCATTGTCGTGACG 3′) and the newly designed primer Pan5.8S-239R (5′ GCCGAGAGTCGTGTGGTTTA 3′). The forward primer spans the 18S/ITS1 border, and the reverse primer spans the ITS1/5.8S border. For ITS2 we used a combination of primers Pan5.8S-316F (5′ ACCATCGAGTCTTTGAACGCA 3′) and ITS2_582R (5′ AAAGGGTCCATTGAGGCCAT 3′). Because the forward primer is located in the 5.8S gene, the amplified ITS2 region included 51 nt (excluding the primer) of the 5.8S gene. The primers were tagged with 12-bp barcodes at the 5′ end. Each sample was PCR-amplified with a unique combination of barcoded primers to facilitate multiplexing prior to sequencing. For both spacers, we performed the PCRs in the total volume of 25 µl containing 1× concentrated Phusion HF buffer (New England Biolabs, Ipswich, MA, USA), 2.5 mM of MgCl_2_, 0.2 mM of each dNTP, 0.2 µM of each primer, 0.4 units of Phusion High Fidelity Polymerase (New England Biolabs) and 1–5 ng of genomic DNA or 1 µl of cDNA diluted 1:10. We performed the PCRs in an Eppendorf Mastercycler Pro S (Eppendorf, Hamburg, Germany) using the following cycling conditions: initial denaturation for 30 s at 98°C, followed by 35 cycles of 98°C/10 s, 50°C/30 s (for ITS1 with both DNA and cDNA and ITS2 with DNA as template) or 58°C/30 s for ITS2 with cDNA as template), 72°C/30 s and a final elongation step at 72°C for 10 min. Each sample was amplified in three independent reactions and mixed equimolarly to reduce PCR bias. The mixed PCR products of each sample were purified using the SPRI select paramagnetic beads (Brea, CA, USA) to remove primer dimers. The concentration of each sample was measured using a Qubit fluorometer with a broad-range dsDNA assay kit (Thermo Fisher Scientific). The PCR products of each spacer/template combination were pooled and used for sequencing libraries. The sequencing libraries were prepared following the protocol of Belyayev et al., 2019, except that 300 ng of the pooled PCR product was used as a template and that sonication was not performed. The libraries were checked for adaptor contamination using BioAnalyzer 2100 (Agilent, Santa Clara, CA, California) at the OMICS-Genomics Laboratory of the BIOCEV (Vestec, Czech Republic) and sequenced on the Illumina MiSeq platform with 2 × 300-bp pair-end settings at Macrogen Europe (Amsterdam, Netherlands).

### rDNA sequence analysis

Analysis of rDNA sequences involved the following steps: (1) development of a reference database needed for steps 2 and 3, (2) preprocessing of reads and sequence filtering, and (3) analysis of the final dataset, that is, assigning the sequences to parental types.

### Development of the database and estimation of sequence divergence among parental taxa

To gain insight into interspecific variation in the ITS region of parental taxa, we amplified and sequenced the entire ITS1-5.8S-ITS2 region from all twelve individuals of parental species as described in Mahelka et al. (2007). The ITS sequences have been deposited in the GenBank repository under the accession numbers OQ346359–OQ346370. Additional 26 sequences representing the species were downloaded from the GenBank repository, aligned together and used as a reference database for the subsequent data analysis (**Supplementary File 1**). Next, we estimated average distances between the species using the ‘Between group mean distance’ command with a pairwise deletion option for gaps/missing data, implemented in MEGA11 (Tamura et al., 2021). Diploid and tetraploid *F. pratensis* did not differ in their sequences and were therefore considered a single species. The estimated mean sequence distances between species for ITS1 and ITS2, respectively, are as follows: *F. pratensis/F. glaucescens* – 0.107 and 0.043, *F. glaucescens*/*L. multiflorum* – 0.111 and 0.073, *F. pratensis*/*L. multiflorum* – 0.017 and 0.033, and *F. pratensis*/*L. perenne* – 0.020 and 0.041. Thus, the mean interspecific divergence ranged between 1.7% to 11.1%.

### Preprocessing of reads and sequence filtering

After quality checking, forward and reverse reads were joined, and the resulting sequences belonging to individual samples were demultiplexed using the *make.contigs* command as implemented in mothur v. 1.43.0 (Schloss et al., 2009). We applied this procedure for each of the four libraries separately. Afterwards, we merged the cDNA and DNA sequences of the same ITS region into a single fasta file. From this point onward, we processed two datasets in parallel: (1) the ITS1 dataset containing ITS1 sequences amplified from both DNA and cDNA, and analogically (2) the ITS2 dataset. We used *mothur* to align the sequences with the reference database to filter out sequences not matching the reference, for example those that may be the result of contamination by endophytes, or chimeric or otherwise unspecific sequences. This approach generated two files: a fasta file containing all unique sequences identified in the entire dataset and a table with the abundance of the sequences in each sample.

### Assigning the sequences to parental types

Classification of the sequences, that is, their assignment to their parental types, was done using the stand-alone version of BLAST with the reference alignment used as a database. Thus, all sequences were Blasted with default settings against the database, and only the best hit with a length greater than 200 nt was kept for each query. Next, we discarded all sequences matching the best hit with a lower identity than the set threshold. The thresholds were different for ITS1 and ITS2, and were set based on the most closely related species pair (species pair with the lowest divergence). For both ITS1 and ITS2, the *F. pratensis*/*L. multiflorum* species pair showed the lowest divergence (0.017 for ITS1 and 0.033 for ITS2). The thresholds were set slightly above the divergence values to avoid cross-matches. Thus, in reality, we kept only sequences matching any sequence from the database with an identity greater than 98.5% in the ITS1 dataset and 97% in the ITS2 dataset. To be consistent, we kept these thresholds for all crosses, generations and genotypes. Hits below these thresholds were regarded as ambiguously classified sequences and could not be analysed further. As a next step, we removed all singletons, that is, sequences found only once in the entire dataset. These may represent artificial sequence polymorphism due to polymerase errors or other PCR artefacts.

The above-described process was applied to each cross and its parental taxa. As an outcome, the proportion of parental sequences at the genomic (DNA samples) and transcriptomic (cDNA samples) levels was obtained. The mean values with standard deviations were calculated from biological replicates data (different individuals) and visualized in bar charts.

### Inference of rDNA homogenization in the *F. pratensis* (4x) × *L. multiflorum* (4x) hybrids

We used the sequence diversity of rDNA as an estimate of rDNA homogenization. To account for this sequence diversity, we estimated the proportion of unique sequence types (hereafter called ribotypes) in each sample by dividing the number of ribotypes by the total number of sequences. In this calculation we encountered a significant correlation between the number of ribotypes and the total number of sequences in each sample (pooled DNA and cDNA datasets; R = 0.73, p < 2.2*×*10^−16^). To avoid the bias caused by differences in the total number of sequences obtained for each sample, we subsampled the datasets. Thus, we used the same number of subsampled sequences for all genotypes within each dataset (334 for ITS1 and 370 for ITS2), and this number corresponded with the second lowest number of sequences obtained for any genotype within the respective dataset. A comparison of sequence diversity can be done at any hierarchical level, that is, among samples, species, generations, ITS regions (markers) or different templates (DNA vs cDNA). Specifically, we asked if there is a difference in rDNA diversity among particular generations of *F. pratensis* (4x) × *L. multiflorum* (4x) hybrids (including parents).

## Results

### Physical localization of 45S rDNA loci in parental species and hybrids

**Figure 1:**
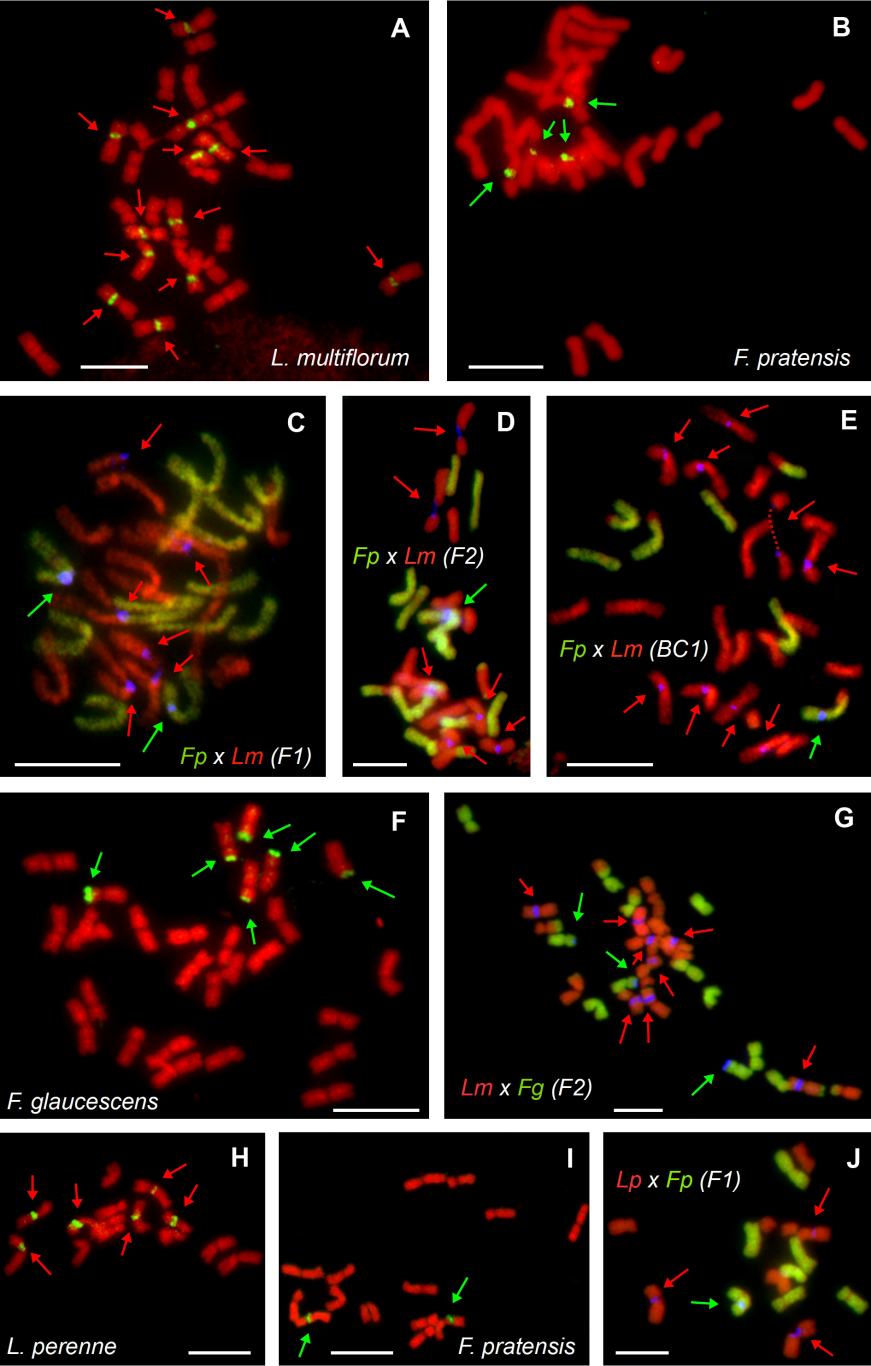
Molecular cytogenetic analysis of parental species and hybrids within the *Festuca–Lolium* complex. FISH on mitotic metaphase plates of autotetraploid *Lolium multiflorum* **(A)**, autotetraploid *Festuca pratensis* **(B)**, their allotetraploid *F. pratensis × L. multiflorum* hybrids in F_1_ **(C)**, F_2_ **(D)** and BC_1_ **(E)** generations, tetraploid *F. glaucescens* **(F)**, F_2_ hybrid of *L. multiflorum × F. glaucescens* **(G)**, diploid *L. perenne* **(H)**, diploid *F. pratensis* **(I)** and their homoploid (diploid) *L. perenne × F. pratensis* hybrids of the F_1_ generation **(J)**. *In situ* hybridization was performed using a probe for 45S rDNA-labelled with biotin (green pseudocolour on **A, B, F, H, I** or blue pseudocolour on **C–E, G, J**), total genomic DNA of *F. pratensis* **(C-E, J)** or *F. glaucescens* **(G)** labelled with FITC and used as a probe (green colour) and genomic DNA of *L. multiflorum* used as blocking DNA (red pseudocolour; **C–E, G, J**). Chromosomes were counterstained using DAPI (red pseudocolour). Loci of 45S rDNA are marked based on their origin, which was either the *Festuca* (green arrows) or the *Lolium* (red arrows) genome. Note that each of the hybrids has 45S rDNA loci from both parents, even though their proportion varies. In **(E)**, one of the chromosomes is broken in secondary constriction (dashed line). Scale bar, 10 µm.

Genomic *in situ* hybridization was carried out to determine the genomic composition of the hybrids. Using fluorescent *in situ* hybridization, we were able to quantify the number of 45S rDNA loci and assign them to the particular parental genomes in a subset of investigated plants (**Table 2**). In tetraploid *L. multiflorum*, there were twelve interstitial 45S rDNA loci located on chromosomes 2L, 3L and 7L (**Figure 1A**). This observation is consistent with our previous analysis (Kopecký et al., 2010). Six interstitial 45S rDNA loci were observed on the same chromosomes in diploid *L. perenne* (**Figure 1H**). In *F. pratensis*, two interstitial 45S rDNA loci were observed on chromosome 3F in the diploid genotype, and four loci were observed in tetraploids (**Figures 1B, I**; Kopecký et al., 2010; Křivánková et al., 2017). Six 45S rDNA loci were located in terminal chromosome regions in allotetraploid *F. glaucescens* (**Figure 1F**).

**Table 2:** Cytogenetic characterization of Festulolium (*Festuca* × *Lolium*) and *F. glaucescens × F. pratensis* hybrids and their parental species.

Cross, parent	Genotype	Generation	Genome composition	rDNA loci		
				Lolium-type	Fp-type	Fg-type
Fp (4x) × Lm (4x)	FL_06	F1	14Lm+14Fp			
	FL_07	F1	14Lm+14Fp			
	FL_08	F1	14Lm+14Fp	6 interstitial	2 interstitial	
	FL_09	F1	14Lm+14Fp	6 interstitial	2 interstitial	
	FL_10	F1	14Lm+14Fp			
	FL_18	F2	14Lm+13Fp	6 interstitial	1 interstitial	
	FL_19	F2	n.a.			
	FL_26	F3	n.a.			
	FL_20	BC	n.a.			
	FL_21	BC	21Lm+7Fp	8 interstitial	1 interstitial	
	FL_22	BC	21Lm+6Fp	9 interstitial	1 interstitial	
	FL_23	BC	n.a.			
	FL_24	BC	20Lm+6Fp	8 interstitial	1 interstitial	
	FL_25	BC	n.a.			
Lm (4x) × Fp (4x)	FL_11	F1	14Lm+14Fp			
	FL_12	F1	14Lm+14Fp			
Lp (2x) × Fp (2x)	FL_13	F1	7Lp+7Fp	3 interstitial	1 interstitial	
	FL_14	F1	7Lp+7Fp	3 interstitial	1 interstitial	
	FL_15	F1	7Lp+7Fp	3 interstitial	1 interstitial	
	FL_16	F1	7Lp+7Fp	3 interstitial	1 interstitial	
	FL_17	F1	7Lp+7Fp			
Lm (4x) × Fg (4x)	FL_27	F2	n.a.			
	FL_28	F2	14Lm+14Fg	6 interstitial		2 terminal
	FL_29	F2	16Lm+12Fg	8 interstitial		3 terminal
	FL_30	F2	16Lm+12Fg	6 interstitial		2 terminal
	FL_41	F2	16Lm+12Fg	7 interstitial		2 terminal
Fg (4x) × Fp (4x)	FL_01	F1	14Fg+14Fp			
	FL_02	F1	14Fg+14Fp		2 interstitial	3 terminal
	FL_03	F1	14Fg+14Fp			
	FL_04	F1	14Fg+14Fp			
	FL_05	F1	14Fg+14Fp			
Lm (4x) [Fp × Lm]	FL_37	Parent_Lm	28 Lm	12 interstitial		
	FL_42	Parent_Lm	28 Lm	12 interstitial		
	FL_43	Parent_Lm	28 Lm	12 interstitial		
Fp (4x) [Fp × Lm]	FL_34	Parent_Fp	28 Fp		4 interstitial	
	FL_35	Parent_Fp	28 Fp		4 interstitial	
	FL_36	Parent_Fp	28 Fp		4 interstitial	
	FL_39	Parent_Fp	28 Fp		4 interstitial	
Fg (4x) [Lm × Fg]	FL_31	Parent_Fg	28 Fg			6 terminal
	FL_32	Parent_Fg	28 Fg			6 terminal
	FL_33	Parent_Fg	28 Fg			6 terminal
Lp (2x) [Lp × Fp]	FL_38	Parent_Lp	14 Lp	6 interstitial		
Fp (2x) [Lp × Fp]	FL_40	Parent_Fp	14 Fp		2 interstitial	

For a subset of genotypes used in this study, the genomic composition and physical localization of parental 45S rDNA loci were determined. For the parental species it is written in square brackets for which crosses they were used. Lm, *Lolium multiflorum*; Lp, *L. perenne*; Fp, *Festuca pratensis*; Fg, *F. glaucescens*. n.a., not analysed.

All the *Festuca × Lolium* and *F. glaucescens × F. pratensis* hybrids investigated harbored at least one 45S rDNA locus inherited from each parent. In both investigated F_1_ tetraploid *F. pratensis × L. multiflorum* hybrids, there were two interstitial 45S rDNA loci on *F. pratensis* chromosomes and six interstitial 45S rDNA loci on *L. multiflorum* chromosomes (**Figure 1C**). One F_2_ plant of the same cross combination (FL_18, **Figure 1D**) had 13 F*. pratensis* chromosomes (two with homeologous translocations) and 14 *L. multiflorum* chromosomes (four with homeologous translocations). In this genotype, there were six interstitial 45S rDNA loci on *L. multiflorum* chromosomes and a single interstitial 45S rDNA locus on one *F. pratensis* chromosome (**Figure 1C**). The missing *Festuca* chromosome has to be 3F, as there was only a single locus of 45S rDNA. We further investigated three plants of the BC_1_ generation. One plant (FL_21) had 21 *Lolium* + 7 *Festuca* chromosomes with eight 45S rDNA loci residing on *Lolium* chromosomes and one 45S rDNA locus residing on a *Festuca* chromosome. This suggests that one 45S rDNA-bearing chromosome of *Lolium* was replaced by one 45S rDNA non-bearing chromosome. Another plant (FL_22; **Figure 1E**) had 21 *Lolium* + 6 *Festuca* chromosomes, and harbored nine loci and one locus of rDNA, respectively. This suggests that one missing *Festuca* chromosome is not chromosome 3F bearing 45S rDNA. The third plant (FL_24) had 20 *Lolium* + 6 *Festuca* chromosomes, altogether with eight loci and one locus of rDNA, respectively. This suggests that one missing *Lolium* chromosome is either 2L, 3L, or 7L and that one missing *Festuca* chromosome is not the 3F chromosome.

Four plants of F_2_ *L. multiflorum × F. glaucescens* hybrids were investigated. One plant (FL_28) had 14 *Festuca* + 14 *Lolium* chromosomes with two and six 45S rDNA loci, respectively. This suggests that one 45S rDNA-bearing chromosome was replaced by one 45S rDNA non-bearing chromosome. Another plant (FL_29; **Figure 1G**) had 12 *Festuca* chromosomes with three 45S rDNA loci and 16 *Lolium* chromosomes with eight 45S rDNA loci. This suggests that two substituted (missing) *Festuca* chromosomes were non-bearing 45S rDNA, and both additional *Lolium* chromosomes were 45S rDNA bearing. This may suggest that at least some 45S rDNA-bearing chromosomes from both species (*L. multiflorum* and *F. glaucescens*) are not homeologues. In the remaining two plants (FL_30 and FL_41), there were two 45S rDNA loci residing on 12 *Festuca* chromosomes, indicating that one out of these two missing chromosomes was 45S rDNA-bearing and the other 45S rDNA non-bearing. There were 16 *Lolium* chromosomes with six and seven 45S rDNA loci, indicating that, consequently, no chromosome and one chromosome above the standard 14-chromosome karyotype was a 45S rDNA-bearing chromosome, respectively. Even though the location of the 45S rDNA loci seems to be the same in hybrids and their parental species, we cannot rule out the possibility that some of the loci had been translocated in F_2_ and BC hybrids, a phenomenon occasionally seen in interspecific plant hybrids.

Four diploid F_1_ *L. perenne × F. pratensis* hybrids were analyzed. All of them had 7 *Lolium* + 7 *Festuca* chromosomes with three and one 45S rDNA loci, respectively (**Figure 1J**). Additionally, we analysed a single genotype of *F. glaucescens × F. pratensis* hybrids (FL_02). It harbored 14 F*. pratensis* + 14 F*. glaucescens* chromosomes with the number of 45S rDNA loci corresponding to the sum of the haploid parental sets, that is, two from autotetraploid *F. pratensis* and three from tetraploid *F. glaucescens* (**Table 2**).

### Inference of genome dominance in *L. multiflorum* × *F. glaucescens* and *F. glaucescens* × *F. pratensis* hybrids

We used GISH to determine the genomic composition of successive generations of *L. multiflorum × F. glaucescens* and *F. glaucescens × F. pratensis* hybrids. We observed that the genome composition shifted between the F_1_ and F_2_ generations from 14 *Lolium* + 14 *Festuca* chromosomes to 14.72 *Lolium* + 12.79 *Festuca* chromosomes in *L. multiflorum × F. glaucescens* hybrids (72 plants investigated) and from 14 *F. pratensis* + 14 *F. glaucescens* to 14.00 *F. pratensis* + 13.56 *F. glaucescens* chromosomes in *F. glaucescens × F. pratensis* hybrids (9 plants investigated). This data indicates *L. multiflorum* and *F. pratensis* to be the dominant genomes in the aforementioned hybrids, respectively.

### rDNA sequence characteristics and classification

Following the filtering of the sequences, the final dataset, including the sequences amplified from DNA as well as cDNA and from both the ITS1 and ITS2 regions, consisted of 2,508,260 sequences generated from 43 samples (12 of parental species and 31 of hybrids). Using Blast against a reference database, all sequences were assigned to either *Festuca*- or *Lolium*-type (alternatively to *F. glaucescens*- or *F. pratensis*-type in *F. glaucescens × F. pratensis* genotypes) using a threshold set based on the difference of parental ITS sequences. The number of sequences generated for particular genotypes, and their attribution to parental types, are presented in **Supplementary Table 1**. The datasets of ITS1 and ITS2 sequences were analysed separately because they originated from separate PCRs. rDNA expression was expressed as the relative representation of parental ribotypes in particular genotypes.

### Abundance and expression patterns of rDNAs in parental species

**Figure 2:**
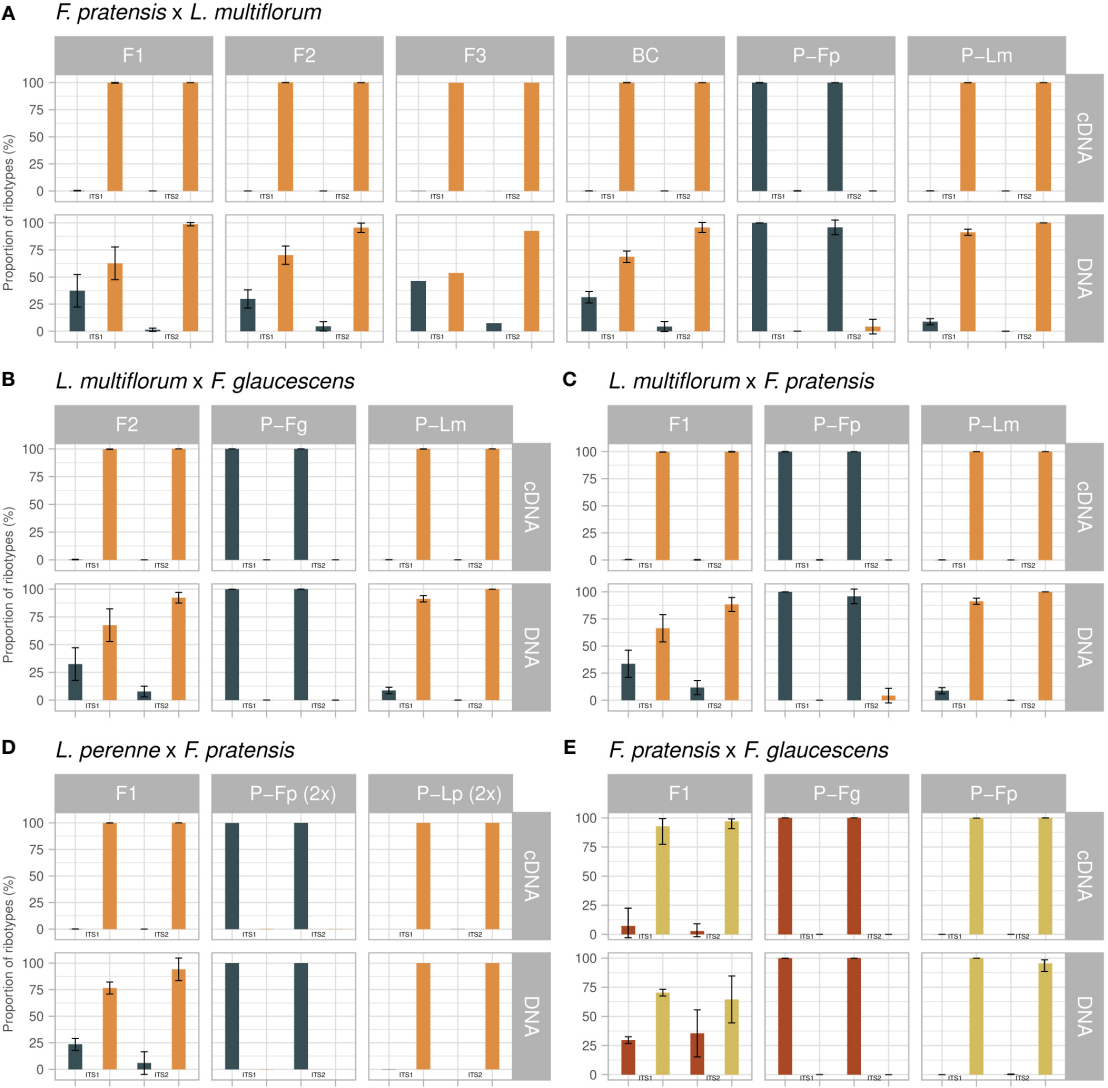
Expression analysis of rRNA in Festulolium (Festuca × Lolium, **A–D**) and F. glaucescens × F. pratensis **(E)** hybrids. For each cross and generation, the relative quantification of parental types (percent of sequences, mean value) is presented for DNA and cDNA templates and for the ITS1 and ITS2 regions. P, parental species; Lm, Lolium multiflorum; Lp, L. perenne; Fp, Festuca pratensis; Fg, F. glaucescens. Error bars, standard deviation.

The parental genotypes of *Lolium* contained a portion of DNA sequences that corresponded to *Festuca*, and *vice versa*, the parental genotypes of *Festuca* contained a portion of sequences corresponding to *Lolium.* This phenomenon was observed in both the ITS1 and the ITS2 dataset (**Figure 2**; **Supplementary Table 1**). In ITS1, DNA sequences of *L. multiflorum* (4x) contained on average 8.72% of sequences corresponding to a *Festuca*-type sequence whereas in *L. perenne* (2x) the proportion of *Festuca*-type sequences was only 0.08%. In ITS2, the proportion of *Festuca*-type sequences in *L. multiflorum* was 0.01%, and there was none in *L. perenne*. In *Festuca* species, both diploid and tetraploid *F. pratensis* DNA-ITS1 sets contained on average 0.06% of *Lolium*-type sequences, while it was 0.05% in tetraploid *F. glaucescens*. In the DNA-ITS2 set, the tetraploid *F. pratensis* contained on average 4.23% of *Lolium*-type sequences; in *F. glaucescens* this number was 0.04% and diploid *F. pratensis* contained no *Lolium*-type sequences.

Only marginal portions of counterpart sequences were observed in cDNAs. The ITS1 dataset of *L. multiflorum* contained 0.08% of *Festuca*-type sequences, and these were absent in *L. perenne*. In ITS2, both *L. multiflorum* and *L. perenne* contained 0.01% of *Festuca*-type sequences. Similarly to the *Festuca*-in-*Lolium* cases, only a rare occurrence of *Lolium*-type sequences was found in cDNA datasets of fescues. Specifically, 0.20%, 0.11% and 0.01% of *Lolium*-type sequences were found in diploid and tetraploid *F. pratensis* and *F. glaucescens* cDNA-ITS1 sequence sets, while cDNA-ITS2 sequence sets contained 0.04%, 0.05% and 0.02% of *Lolium*-type sequences, respectively. The low proportions of counterpart sequences found in parental cDNAs, corresponding in absolute counts to between zero to ten sequences out of thousands of sequences per genotype, raises the question of whether these are true, biologically relevant ITS copies, or an artefact (see Discussion).

### rDNA expression in hybrids

#### *Festuca pratensis* (4x) × *Lolium multiflorum* (4x) (F_1_, F_2_, F_3_, BC_1_)

This hybrid combination was the only one for which more than one generation was available. At the level of DNA, there was an excess of *Lolium*-type sequences in all four generations and genotypes studied (**Figure 2**; **Supplementary Table 1**). Their average proportions in the ITS1 region were 62.7% (F_1_), 70.2% (F_2_), 53.7% (F_3_) and 68.7% (BC_1_). The ITS2 showed more profound differences, with the following proportions of *Lolium*-type sequences: 98.7% (F_1_), 95.5% (F_2_), 92.5% (F_3_) and 95.7% (BC_1_). A certain excess of *Lolium*-type sequences is expectable due to the 6:2, 6:1, and 8–9:1 ratios of *Lolium*- and *Festuca*-origin rDNA loci in the F_1_, F_2_ and BC_1_ generations, revealed by FISH, respectively (**Figures 1C–E**; **Table 2**). The analysis of the distribution of rDNA loci in F_3_ using FISH was not performed. While the excess of *Lolium*-type sequences can partly be explained by the excess of *Lolium*-derived rDNA loci, the sizes of particular loci are another major factor underlying this phenomenon. Visual observations did not reveal substantial length differences between the loci located on *Festuca* and *Lolium* chromosomes in Festulolium hybrids.

Expression analysis showed that only a few *Festuca*-type sequences were expressed in this hybrid, pointing to ND by the *Lolium*-derived homeologues. This observation was consistent in both the ITS1 and ITS2 datasets. The proportion of *Lolium*-type cDNA sequences was approaching 100% in all generations (> 99.7%). In absolute numbers, there were only a few *Festuca*-type sequences among thousands, or tens of thousands, of *Lolium*-type sequences (**Figure 2**; **Supplementary Table 1**).

#### *Lolium multiflorum* (4x) × *Festuca pratensis* (4x) (F_1_)

This cross was reciprocal to the previous one. Again, there was an excess of *Lolium*-type sequences in DNA in both genotypes. ITS1 showed a 66.4% vs 33.6% ratio, and ITS2 showed an 88.3% vs 11.7% ratio in favor of *Lolium*-type (**Figure 2**; **Supplementary Table 1**). *Lolium*-type sequences clearly prevailed in cDNA, where they accounted for almost 100% of all sequences (99.6% and 99.8% in ITS1 and ITS2, respectively). In this hybrid, none of the genotypes were analysed using *in situ* hybridization for rDNA loci number and localization. However, as all other F_1_ hybrids displayed a halved sum of the rDNA loci from each parent, we expect this hybrid to follow this pattern and to possess six loci from *L. multiflorum* and two loci from *F. pratensis*.

#### *Lolium multiflorum* (4x) × *Festuca glaucescens* (4x) (F_2_)

Average values, calculated for the five genotypes of the F_2_ generation, showed very similar results to the above-mentioned crosses. At the level of DNA, *Lolium*-type sequences exceeded *Festuca*-type sequences in a ratio of 67.5% vs 32.5% for ITS1 and 91.0% vs 9.0% for ITS2 (**Figure 2**; **Supplementary Table 1**). The observed ratios of *Lolium*- and *Festuca*-derived rDNA loci were 6:2, 7:2, or 8:3 in three genotypes (**Figure 1G**; **Table 2**). As in the previous hybrids, we observed an almost 100% predominance of the *Lolium*-type sequences in cDNA (99.7% for ITS1 and 99.99% for ITS2).

#### *Lolium perenne* (2x) × *Festuca pratensis* (2x) (F_1_)

This was the only cross involving diploid genotypes. As in the tetraploids, *Lolium*-type sequences in DNA prevailed over those corresponding to *Festuca*. In ITS1, the observed average ratio of 76.5% vs 23.5% (**Figure 2**; **Supplementary Table 1**) well matched the number of rDNA loci showing a 3:1 ratio in favor of *Lolium*-type (**Figure 1J**; **Table 2**). DNA-ITS2 showed an increased proportion of *Lolium*-type sequences, as observed in the previous hybrids (94.1% vs 5.9%). In the cDNA dataset, we detected only 19 *Festuca*-type sequences out of 138,881 sequences, demonstrating almost 100% expression of *Lolium*-derived sequences in this hybrid (99.9% for ITS1 and 100% for ITS2).

#### *Festuca glaucescens* (4x) × *Festuca pratensis* (4x) (F_1_)

This hybrid was the only one between species of the same genus. In this cross, we did not observe a marked discrepancy between the ITS1 and ITS2 datasets (**Figure 2**; **Supplementary Table 1**). Here, *F. pratensis*-type sequences prevailed over the type corresponding to *F. glaucescens*: The average ratio for five genotypes was 70.3% vs 29.6% in ITS1 and 62.4% vs 37.6% in the ITS2 dataset. Such ratios are the opposite of what one would expect solely based on the ratio of rDNA loci (2 loci derived from *F. pratensis* and 3 loci from *F.* glaucescens; **Table 2**). However, as noted above, the exact number of rDNA copies is unknown. A minor proportion of sequences corresponded to a *Lolium*-type, ranging between 0.02% and 0.07%. In this hybrid, both parental types appeared in cDNA. Namely, in ITS1 the proportion of sequences was 92.7% vs 7.2% in favor of the *F. pratensis* type, while in ITS2 the ratio was 96.6% vs 3.4%. *Lolium*-type sequences in cDNA accounted for 0.01% and 0.08% in ITS1 and ITS2, respectively.

### Inference of rDNA homogenization in *F. pratensis* (4x) × *L. multiflorum* (4x) hybrids

**Figure 3:**
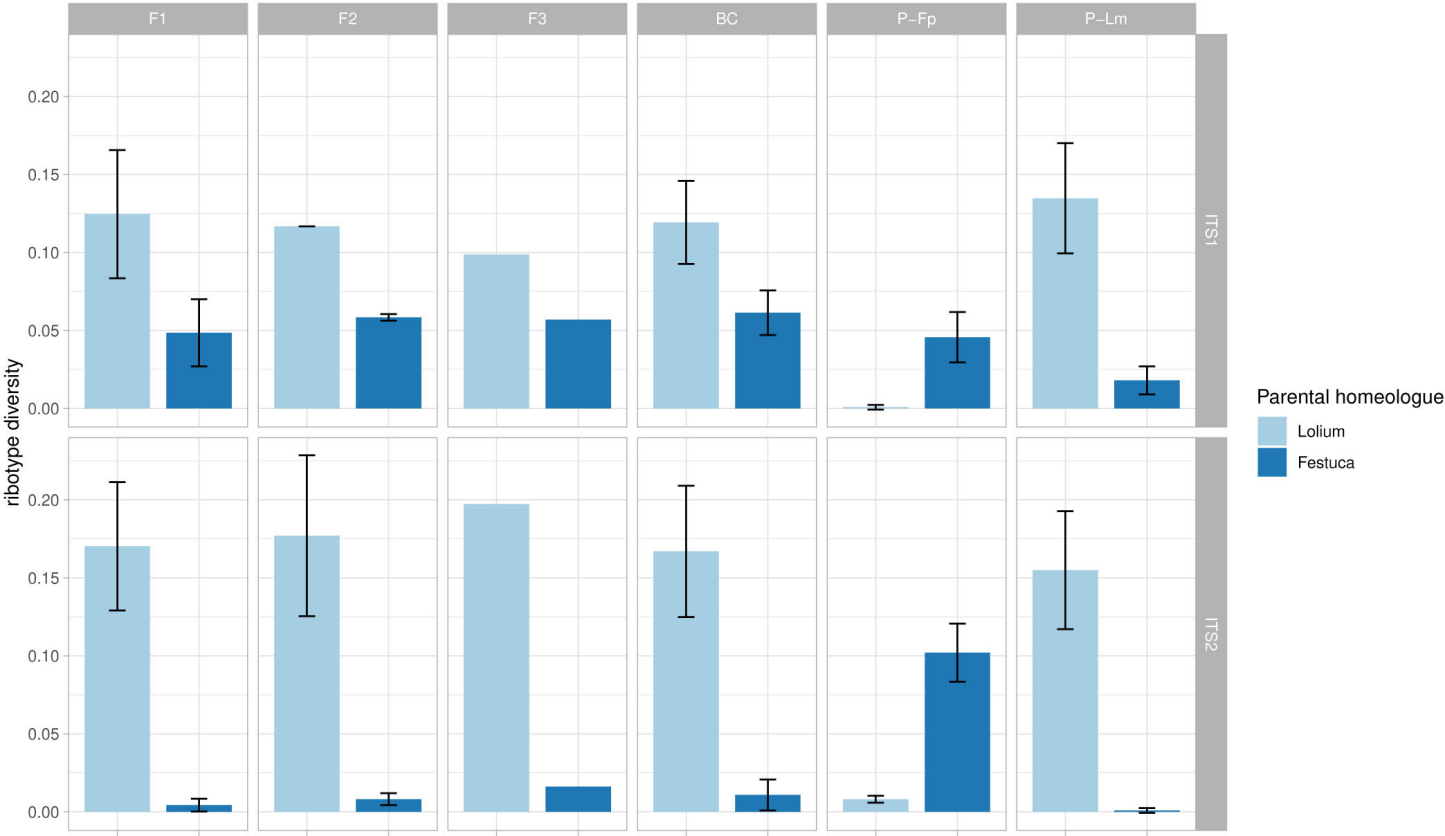
rDNA diversity of *Festuca*-type and *Lolium*-type homeologues in multiple generations of *F. pratensis* × *L. multiflorum* hybrids. Ribotype diversity was calculated as the number of unique sequence types (ribotypes) divided by the total number of sequences in each sample. Bars represent average ribotype diversity for *Lolium*-type (light blue) and *Festuca*-type (dark blue) homeologues across generations and their parents, and values for both ITS1 and ITS2 markers are given. Error bars, standard deviation. No significant differences among generations were observed using ANOVA (for details see text).

Ribotype diversity, calculated as the number of ribotypes divided by the number of sequences (subsampled) in each sample, still showed a correlation with the total number of sequences generated for each sample (i.e. the original dataset before subsampling). However, this correlation was much weaker than the original correlation resulting from non-subsampled data (R = 0.28, p = 0.00013 vs R = 0.73, p < 2.2*×*10^−16^; **Supplementary Figure 1**). Neither the *Lolium*-type nor the *Festuca*-type homeologue showed clear signatures of DNA sequence homogenization among particular generations in the *F. pratensis* (4x) × *L. multiflorum* (4x) hybrid (**Figure 3**; ANOVA: ITS1, *Festuca*-homeolog: F = 0.541, p = 0.665, *Lolium*-homeologue: F = 0.185, p = 0.904; ITS2, *Festuca*-homeologue: F = 1.092, p = 0.401, *Lolium*-homeologue: F = 0.151, p = 0.927). These data indicate an absence of rDNA homogenization in the initial generations of this cross.

### Nucleolar dominance in Festulolium hybrids

Overall, the data obtained in this study show that ND in leaf tissue occurs in all hybrid combinations between *Festuca* and *Lolium* species. In all interspecific crosses and genotypes, *Festuca*-type sequences were nearly absent from cDNA, demonstrating a strong, nearly 100% (99.62–99.99%, **Figure 2**; **Supplementary Table 1**) dominance of *Lolium*-type sequences. In this respect, ND in Festulolium manifests itself by the same dominant genome (*Lolium*) as observed in studies investigating genomic dominance (Zwierzykowski et al., 2006; Majka et al., 2023; results of this study). To sum up, the following specific observations were made in *Festulolium* hybrids: (1) ND in leaf tissue occurred in all interspecific hybrids, generations and genotypes investigated, and consistently displayed nearly 100% dominance of *Lolium* homeologues; (2) the onset of ND was immediate, manifested already in the F_1_ generation; (3) ND occurred irrespective of the ploidy level; (4) maternity did not have any effect on ND in Festulolium; ND was observed in reciprocal crosses at a similar level; (5) no significant between-generation homogenization of rDNA has been observed in *F. pratensis* (4x) × *L multiflorum* (4x) hybrid; and (6) in the *F. pratensis* × *F. glaucescens* (F_1_) hybrid, we observed the expression of both parental homeologues, although upregulation of *F. pratensis*-type genes was recorded. Also in this hybrid, the dominance of *F. pratensis* rDNA is in line with the whole-genome dominance of *F. pratensis*.

## Discussion

The aim of this study was to find out if hybrids showing dominance at the whole-genome level (i.e. genome dominance at chromosome level) also show dominance in the expression of ribosomal DNA (i.e. nucleolar dominance, ND) and, if so, whether the dominant genome is the same. For this purpose, we used Festulolium hybrids for which genome dominance with *Lolium* as the dominant genome was described previously (Kopecký et al., 2006; Zwierzykowski et al., 2006; Glombik et al., 2021; Majka et al., 2023). In addition to Festulolium, we tested the same hypothesis in a hybrid combination between *Festuca glaucescens* and *F. pratensis*, the two genomes that are submissive in Festulolium hybrids.

### Limitations of the study

Although we believe that the results presented in this study are solid and clearly support the conclusions outlined, we are aware that they have some limitations arising from both the nature of the plant material available and the nature of the dataset itself.

#### Prevalence of *Lolium*-type rDNA loci in hybrids

One may speculate that ND of the *Lolium* genome is conditioned by the prevalence of *Lolium*-type rDNA loci (dosage effect, Chen et al., 1998). Indeed, none of the genotypes showed an equal number of rDNA loci or a prevalence of *Festuca*-type loci. However, the disproportion of rDNA loci in hybrids stems from the patterns existing in parental species: While tetraploid *L. multiflorum* harbors six pairs of rDNA loci, there are only three and two pairs of loci in tetraploid *F. glaucescens* and *F. pratensis*, respectively. Diploid *L. perenne* has three pairs, but diploid *F. pratensis* has only one pair. In this respect, it would be interesting to study the ND pattern in a genotype with the number of *Festuca*-type rDNA loci exceeding those of *Lolium*-type. Such maintenance of dominance effect was demonstrated in *Solanum* (Komarova et al., 2004). In a monosomic addition line carrying only one NOR-bearing chromosome of the dominant parent, the dominance remained stable despite the supernumerary rDNA loci of the submissive parent (Komarova et al., 2004). Notwithstanding, it has been shown repeatedly that ND is not an effect of rDNA copy number, as minority ribotypes showed overexpression over those whose rDNA copies largely prevailed, such as in the already mentioned genus *Solanum* and also in *Tragopogon* (Matyášek et al., 2007; Dobešová et al., 2015). Unfortunately, our 2-year effort to obtain a reasonable number of seeds from a backcross of an F_1_ hybrid to *F. pratensis* failed, so we cannot rule out the possibility of a dominant parent switch in ND of *Festuca × Lolium* hybrids. However, in *F. glaucescens × F. pratensis* hybrids, the proportion of rDNA loci of 2:3 in favor of *F. glaucescens* does not correspond to the ratio of cDNA sequences (only 7.2% of ITS1 and 3.4% of ITS2 sequences of *F. glaucescens* origin), indicating that the number of loci is not a critical parameter for the establishment of ND in hybrids of the *Festuca-Lolium* complex.

#### The differential amplification of ITS1 and ITS2 sequences

In all hybrids involving *Festuca* and *Lolium*, we observed a higher ratio of *Lolium*-type sequences in the ITS2 dataset. From the available data, it cannot be said that one or the other dataset better corresponds to reality, because the exact number of rDNA copies is not known. If we were to consider the number of rDNA loci as a proxy for the number of rDNA copies, even then the data are not unambiguous. Whereas ITS1 data better corresponds with the theoretical ratio based on the number of loci in the F_1_ generations, it is the ITS2 dataset which corresponds better in the BC_1_ generation. Contradictory genotype-dependent results were observed in F_2_. However, based on the data from the F_1_ generations, in which the ratios of parental types are closest to balanced, it is likely that certain preferential amplification of *Lolium*-type ITS2 sequences indeed occurred. The reason is unknown, but the absence of this phenomenon (or at least its decreased markedness) in *F. glaucescens × F. pratensis* hybrid suggests that this has nothing to do with the amplification primers alone. To our knowledge, either ITS1 or ITS2 is most often used to infer ND in plants (e.g. Matyášek et al., 2007; Książczyk et al., 2011; Sochorová et al., 2017; Borowska-Zuchowska et al., 2020; Borowska-Zuchowska et al., 2021), and no direct comparison of the two markers is available. In any case, since the main aim of the work was to analyze the sequences qualitatively (i.e. to assign them to a parental type), not quantitatively, and since no discrepancy was found between the ITS1 and ITS2 datasets in this respect (in terms of dominancy), the data from both datasets can be considered valid.

#### Parental species share DNA – an artefact or a true biological phenomenon?

Both parental species contained a portion of sequences that corresponded to their counterparts. From the phylogenetic point of view, all fescues and ryegrasses involved in this study belong to the so-called ‘broad-leaved’ clade of the section Schedonorus and form a paraphyletic group consisting of *Festuca* + *Lolium* + *Micropyropsis tuberosa* species in the ITS tree (Catalán et al., 2004). It therefore cannot be ruled out that different species from this clade share DNA due to common ancestry. However, this scenario is unlikely in our case due to the inconsistent presence of ‘foreign’ ITS types between ITS markers, and among parental species. Namely, while the presence of *Festuca*-type sequences in *Lolium* was mainly detected in ITS1 sequences, *Lolium*-type sequences in *Festuca* were observed in ITS2. Furthermore, a notable portion of *Festuca*-type sequences was found in *L. multiflorum* but not in *L. perenne*, and *Lolium*-type sequences were found in *F. pratensis* but not in *F. glaucescens*. Based on these arguments, we consider the observed pattern an artefact caused by sequencing errors or difficulties in attributing sequences to parental types due to a lower-than-expected similarity of a portion of sequences. Another potential pitfall in molecular biology, namely DNA contamination, can be ruled out in this case, as it would imply consistent admixtures in both ITS1 and ITS2 datasets, which is not the case.

### Nucleolar dominance in Festulolium

Ribosomal RNA is an essential structural component of ribosomes, the sites of protein synthesis. The high demand for ribosomal RNAs needed for ribosome assembly is satisfied through the transcription of numerous copies of rRNA genes. There are hundreds to thousands of rDNA units per genome in grasses (Krak et al., 2021; Tulpová et al., 2022), but not all are transcribed. Thus, ND is the plant’s response to an excess of ribosomal genes, manifesting itself by regulating gene expression according to the current need. In hybrids, ND is more easily determinable than in non-hybrids thanks to the distinctness of ancestral rDNA homeologues. For this purpose, the spacers (ITS1 and ITS2) are the markers of choice due to their accelerated mutation rate compared to highly conserved rRNA genes. We observed almost complete silencing of *Festuca*-type homeologues in *Festulolium* hybrids, meaning that the mechanisms behind ND in Festulolium operate effectively. Such a strict, consistent and immediate dominance, observed across four of the five hybrid combinations (*F. glaucescens × F. pratensis* excluded), four generations and twenty-six genotypes, is not usual in plants.

In an attempt to unravel the mechanisms of ND, studies investigating the patterns of rRNA expression at different scales (populations, genotypes, generations, mutant lines, chromosomes, or different organ tissues) have been carried out in plants, including *Arabidopsis* (Chen et al., 1998; Lewis and Pikaard, 2001; Pontvianne et al., 2012; Mohannath et al., 2016), *Brassica* (Hasterok and Maluszynska, 2000a; Książczyk et al., 2011; Sochorová et al., 2017), *Tragopogon* (Matyášek et al., 2007; Dobešová et al., 2015), *Nicotiana* (Kovařík et al., 2008), *Triticum* (Handa et al., 2018; Tulpová et al., 2022) and *Brachypodium* (Borowska-Zuchowska et al., 2019; Borowska-Zuchowska et al., 2020; Borowska-Zuchowska et al., 2021). Whether ND is a direct consequence of hybridization and polyploidization, and is therefore ubiquitous in hybrids and polyploids, remains unclear. For example, in *Glycine*, preferential expression was absent in synthetic polyploids and in some artificial diploid hybrids (Joly et al., 2004). On the contrary, in most natural allopolyploids with a relatively balanced ratio of homeologues, one homeologue was expressed preferentially, though not absolutely. Similarly, variation in expression dominance was observed among natural populations of *Tragopogon* allotetraploids (Matyášek et al., 2007). Not only were populations with complete dominance, partial dominance and codominance found, but so were also individuals showing a reciprocal pattern of expression in the allotetraploid *T. mirus*. These results may indicate a possible role of environmental conditions, including their interaction with standing genotypic variation, in the establishment of ND in natural populations (Matyášek et al., 2007). The absence of this factor in Festulolium hybrids could at least in part explain the stability of their ND.

The plant material tested in this study is well characterized with respect to its origin. By studying several different generations of artificial crosses, we were able to test how rapid the onset of ND is. Festulolium grasses occur only sporadically in nature (and are sterile; Boller et al., 2020), which hinders the comparison of our data on synthetic crosses with natural ones. In particular, it would be interesting to compare the stability and severity of ND observed in synthetic hybrids with plants from well-established natural populations. In this respect, inconsistent scenarios have been reported in other hybrids. For example, one interesting study system is that of *Arabidopsis suecica*, an allotetraploid hybrid of *A. thaliana* and *A. arenosa* (Chen et al., 1998). In natural *A. suecica*, the rRNA genes of *A. thaliana* are repressed. In synthetic hybrids, stochastic silencing of *A. thaliana* rRNA genes was observed in the F_1_ generation, with epigenetic states showing complete dominance, partial dominance and codominance. ND with one dominant type appeared and became fixed in the F_2_ generation. In the *A. suecica* × *A. thaliana* backcross progeny (with *A. thaliana* used for the backcross), the trend was reversed toward the dominance of *A. thaliana* genes, pointing to a gene or genome dosage effect. Unlike in *Arabidopsis*, congruence between natural and synthetic genotypes was reported in allotetraploid *Brassica napus* (*B. rapa* × *B. oleracea*; Książczyk et al., 2011). Uniparental silencing of *B. oleracea* homeologues appeared already in the F_1_ generation, and the patterns were similar in all tissues including leaves, roots and floral buds. In another study on *B. napus*, Sochorová et al. (2017) made a consistent observation concerning expression patterns in which 95% of tested cultivars exhibited A-genome (*B. rapa*) ND whereas only one cultivar showed codominance. This pattern was consistent with the gene conversion process, perhaps still ongoing, causing gradual replacement of the underdominant units of *B. oleracea* with those of A-genome homeologue (*B. rapa*) in allotetraploid *B. napus*.

In Festulolium, we tested rRNA expression in leaf tissue, although we are aware that tissue-specificity is another level of ND in plants. It has been demonstrated that the patterns of ND are not stable across all plant tissues (Volkov et al., 2007; Borowska-Zuchowska et al., 2023). Some level of tissue specificity has been proved in several genera in which the pattern of expression in multiple tissues was investigated. These include, for example, *Arabidopsis* (Pontes et al., 2007), *Brassica* (Hasterok and Maluszynska, 2000a), *Urochloa* (Santos et al., 2020), *Solanum* (Komarova et al., 2004), *Allium* (Hasterok and Maluszynska, 2000b), *Tragopogon* (Dobešová et al., 2015) and *Brachypodium* (Borowska-Zuchowska et al., 2021). On the other hand, relatively stable patterns of expression were observed in *Triticum aestivum*, only showing differences in relative proportions of dominant subtypes among the different tissues (Tulpová et al., 2022). The number of cases listed above raises the question of whether the tissue-specificity of ND is the rule rather than the exception. The example of *Brachypodium* demonstrates that the deeper we delve into the details of the model under study, the greater the probability that we will make a new discovery. It was long thought that in *B. hybridum* there was a strong uniparental dominance toward the D-genome homeologue in roots and leaves. However, a recent study, investigating ND in a new, previously unexplored genotype, found co-dominant expression of both ancestral homeologues in the root tissue (Borowska-Zuchowska et al., 2021; Borowska-Zuchowska et al., 2023; and references therein). Studies on grasses (Santos et al., 2020; Borowska-Zuchowska et al., 2023), but also on the other models, show that ND is the most stable in leaf tissue. Therefore, we performed our analyses on leaf tissue, where the chance of detecting ND is greatest.

### Nucleolar dominance and rDNA homogenization

We computed ribotype diversity to gain insight into the evolution of rDNA arrays in early generations of Festulolium hybrids. Ribosomal DNA evolves according to the concerted evolution theory of multigene families, when rDNA copies within as well as between loci are homogenized in their sequence via unequal crossing over and gene conversion in meiosis (Eickbush and Eickbush, 2007). It has been suggested that ND contributes to the divergence of rDNA in such a way that the silenced genes, free of selection pressure, more likely accumulate mutations and escape homogenization (Kovařík et al., 2008). ND is a reversible process, unless changes in rDNA arrays are so serious that their function is disabled (Borowska-Zuchowska and Hasterok, 2017). The time required for structural changes in DNA can vary between organisms. For example, while ND may only slow down the homogenization process, leading to gradual pseudogenization of inactive genes and their loss through evolutionary time in *Nicotiana* (Kovařík et al., 2008), instant loss of rDNA copies was recorded in F_1_ hybrids of frogs (*Xenopus*; Michalak et al., 2015) and in the F_2_ and BC_1_ generations in *Armeria* (Fuertes Aguilar et al., 1999), demonstrating a rapid onset of homogenizing mechanisms. In Festulolium, we hypothesize that the effect of ND is such that if a mutation load in rDNA arrays occurs, it will more likely negatively affect underdominant, silenced arrays (i.e. *Festuca*-type). We tested this in multiple generations (F_1_, F_2_, F_3_ and BC_1_) of *F. pratensis* (4x) × *L. multiflorum* (4x) hybrid, for which multiple generations were available. We did not observe any signatures of rDNA homogenization in either of *Lolium*- and *Festuca*-like homeologues, as inferred from relatively stable ribotype diversity among the successive generations (**Supplementary Table 2**, **Figure 3**).

### A link between nucleolar and genomic dominance

There is accumulating evidence that ND in plants is difficult to predict, that it is independent of the maternal effect or the parental rDNA copy number, and that it is reversible, developmentally regulated and dosage-(in)dependent. All this makes ND a challenging phenomenon to study. Despite the wealth of information describing gene silencing mechanisms, the most intriguing aspect of ND – the molecular basis dictating which genes to silence and which to transcribe – remains unclear. Although we did not investigate these mechanisms, placing ND in Festulolium in context with the genome-wide dominance described suggests that both phenomena might be driven by the same mechanisms. However, determining these mechanisms is very complex because genome dominance manifests itself at multiple levels whereas the mechanisms behind these manifestations, and the manifestations as such, are seemingly independent of each other. This is also the case of Festulolium, where we observed two conspicuous manifestations of genome dominance – biased gene expression and unequal elimination of parental chromosomes. Glombik et al. (2021) described that the overall gene expression in Festulolium mirrored more frequently the level of the *Lolium* parent than that of the *Festuca* parent. Interestingly, this was most frequently caused by modified expression of *Festuca* alleles. For example, if the expression of a particular gene was higher in the *Lolium* parent than in the *Festuca* parent, the expression of the *Lolium* allele in the hybrid remained the same as in the *Lolium* parent whereas the *Festuca* allele was overexpressed in the hybrid compared to the *Festuca* parent. This indicates that the *Lolium* genome dominance was at least partially caused by its more efficient trans-acting regulators of gene expression (Glombik et al., 2021). However, these processes did not explain the genome dominance at the chromosome level, specifically a shift in the proportion of parental chromosomes towards *Lolium* in successive generations. We investigated this phenomenon in detail, observing the preferential elimination of *Festuca* chromosomes in male meiosis, likely connected with the silencing of the *Festuca* alleles of two kinetochore genes (*NNF1* and *NDC80*) prior to and during meiotic division (Majka et al., 2023). This silencing likely stemmed from the modification of the spatial architecture in the hybrid nucleus during the cell cycle, as genes located across over half of *Festuca* chromosome 7 were more or less silenced prior to and during meiosis, but not in mitosis. Thus, various levels of genome dominance might be determined by different factors without any necessary interdependence of these phenomena.

To conclude, our study brings new evidence that nucleolar dominance is a phenomenon established early after genome merging in interspecific hybridization and completed already in the F_2_ generation, that it is independent of maternity, and that it is in line with the genome dominance at both the chromosome and the transcriptome level (Glombik et al., 2021; Majka et al., 2023). The dominant genome is always the same (*Lolium* in *Lolium × Festuca* hybrids and *F. pratensis* in *F. glaucescens × F. pratensis* hybrids).

## Data availability statement

The datasets presented in this study can be found in online repositories. The names of the repository/repositories and accession number(s) can be found below: https://www.ncbi.nlm.nih.gov/, PRJNA966787 https://www.ncbi.nlm.nih.gov/genbank/, OQ346359–OQ346370.

## Author contributions

VM: Conceptualization, Data curation, Funding acquisition, Methodology, Supervision, Writing – original draft. DK: Conceptualization, Data curation, Funding acquisition, Writing – review & editing. JM: Writing – review & editing. KK: Conceptualization, Data curation, Methodology, Software, Writing – review & editing.

## Supplementary material

Supplementary Figure 1 Effect of the number of sequences on estimated rDNA (ribotype) diversity. X-axis: the original pools of sequences used for the subsampling. Y-axis: number of sequence types (ribotypes) obtained from the subsample of sequences. The blue area represents the 95% confidence interval.

Supplementary File 1 ITS sequences of *Festuca* and *Lolium* species used as queries (fasta). Sequences OQ346359–OQ346370 are from this study.

Supplementary Table 1 Quantification of sequences analysed in this study. For each genotype, the number of sequences (total and relative) and their attribution to parental types (homeologues) are given for DNA and cDNA templates. The ITS1 and ITS datasets are presented separately on separate sheets.

Supplementary Table 2 rDNA (ribotype) diversity of F. pratensis (4x) × L. multiflorum (4x) hybrids. For each genotype, the diversity of the parental types (homeologues) estimated from the subsampled number of sequences is given for DNA and cDNA templates. For ITS1 and ITS2, 334 and 370 sequences were used for the calculations, respectively. The ITS1 and ITS datasets are presented on separate sheets.

Supplementary Table 3 Barcoding tags used in this study.
