# Supplementary figures and images for: Uniparental expression of ribosomal RNA in ×Festulolium grasses: a link between the genome and nucleolar dominance
Source: Front Plant Sci. 2023 Sep 18;14:1276252. doi: 10.3389/fpls.2023.1276252 (PMC10544908; doi:10.3389/fpls.2023.1276252)

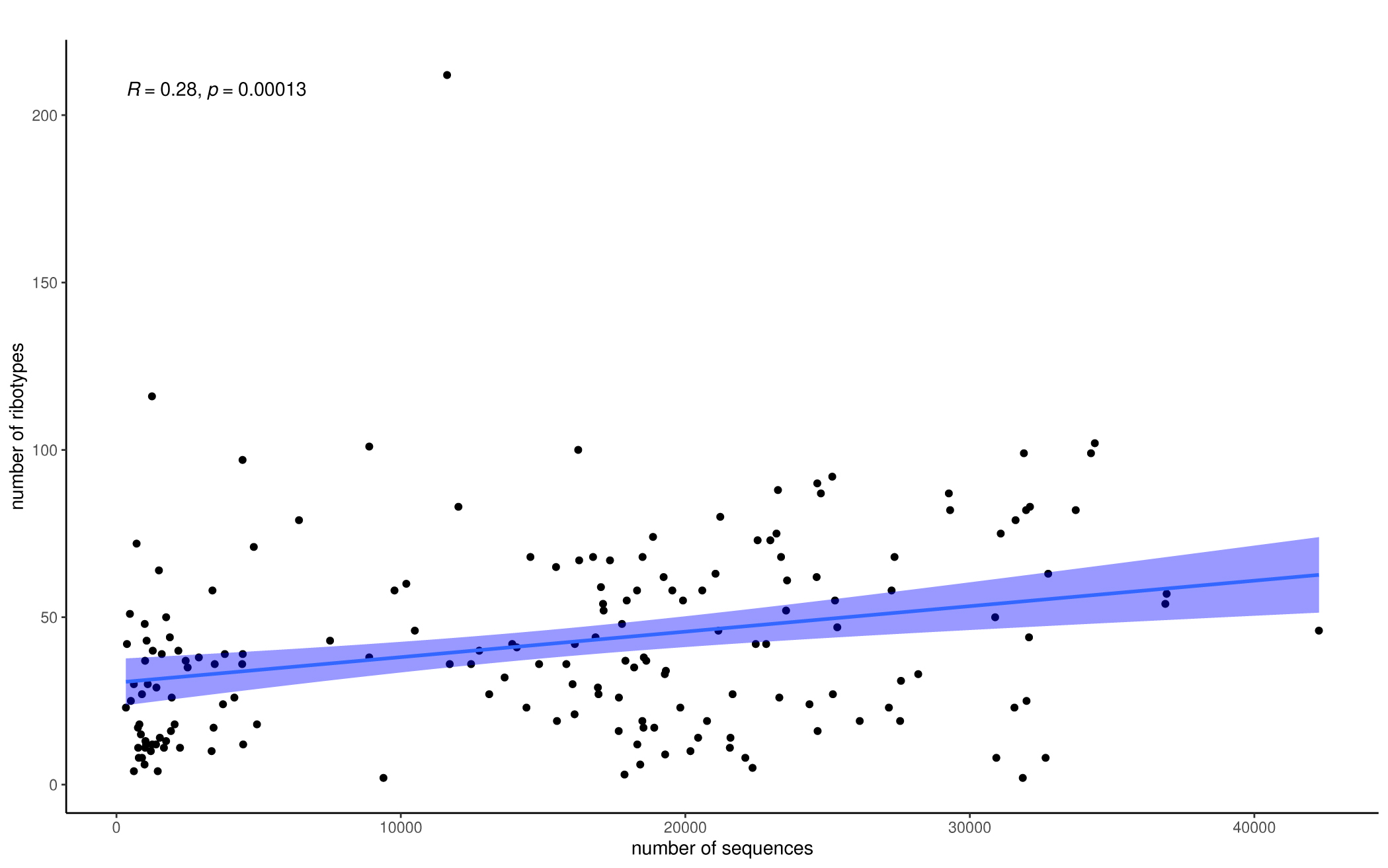

Supplement: Supplementary Figure 1 — Effect of the number of sequences on estimated rDNA (ribotype) diversity. X-axis: the original pools of sequences used for the subsampling. Y-axis: number of sequence types (ribotypes) obtained from the subsample of sequences. The blue area represents the 95% confidence interval. [file Image_1.jpeg]
