# Supplementary material for: Uniparental expression of ribosomal RNA in ×Festulolium grasses: a link between the genome and nucleolar dominance
Source: Front Plant Sci. 2023 Sep 18;14:1276252. doi: 10.3389/fpls.2023.1276252 (PMC10544908; doi:10.3389/fpls.2023.1276252)
Supplement: Supplementary File 1 — ITS sequences of Festuca and Lolium species used as queries (fasta). Sequences OQ346359–OQ346370 are from this study. [file DataSheet_1.docx]

>OQ346359_Festuca_glaucescens_FL_31

CTGACCAAAAAAGACCGCGCACGCGTTATCCAACCTGCTGGGCGGCGGCATCGTCTGCCG

CTTGGCCAAATTCCTCGACAACCTCCCCTCTTCGGAGTTGGGGCTCGGGGTAAAAGAACC

CACGGCGCCGAAGGCGTCAAGGAACACTGTGCCTAACCCGGGGACGCGGTTGGCTTGCTG

ACCGCACCTCGAGTTGCAATGCTATATAATCCACAYGACTCTCGGCAACGGATATCTCGG

CTCTCGCATCGATGAAGAACGTAGCGAAATGCGATACCTGGTGTGAATTGCAGAATCCCG

CGAACCATCGAGTCTTTGAACGCAAGTTGCGCCCGAGGCCTCTTGGCCGAGGGCACGCCT

GCCTGGGCGTCACGCAAAACACGCTCCCAACCCACTAACCTGGTGCGGGACGCGGCATGT

GGCYACCCGTCTCGCAAGGGGCGGTTGGCCGAAGATCCGGCTGCCGGCGTATCGTRCCGG

ACACAGCGCGTGGTAGGCGACCTCGCTTTACTAAACGCAGTGCATCCGGCGCGTAGCCAA

AGCG

>OQ346360_Festuca_glaucescens_FL_32

CTRACCAAAAAAGACCGCGCACGCGTTATCCAACCTGCTGGGCGGCGGCATCGTCTGCCG

CTTGGCCAAATTCCTCGACAACCTCCCCTCTTCGGAGTTGGGGCTCGGGGTAAAAGAACC

CACGGCGCCGAAGGCGTCAAGGAACACTGTGCCTAACCCGGGGACGCGGTTGGCTTGCTG

ACCGCACCTCGAGTTGCAATGCTATATAATCCACAYGACTCTCGGCAACGGATATCTCGG

CTCTCGCATCGATGAAGAACGTAGCGAAATGCGATACCTGGTGTGAATTGCAGAATCCCG

CGAACCATCGAGTCTTTGAACGCAAGTTGCGCCCGAGGCCTCTTGGCCGAGGGCACRCCT

GCCTGGGCGTCACGCAAAACACGCTCCCAACCCACTAACCTGGTGCGGGACGCGGCATGT

GGCYACCCGTCTCGCAAGGGGCGGTTGGCCGAAGATCCGGCTGCCGGCGTATCGTGCCGG

ACACAGCGCGTGGTAGGCGACCTCGCTTTACTAAACGCAGTGCATCCGGCGCGTAGCCAA

AGCG

>OQ346361_Festuca_glaucescens_FL_33

CTGACCAAAAAAGACCGCGCACGCGTTATCCAACCTGCTGGGCGGCGGCATCGTCTGCCG

CTTGGCCAAATTCCTCGACAACCTCCCCTCTTCGGAGTTGGGGCTCGGGGTAAAAGAACC

CACGSCGCCGAAGGCGTCAAGGAACACTGTGCCTAACCCGGGGACGCGGTTGGCTYGCTG

ACCGYACCTCGAGTTGCAATGCTATATAATCCACACGACTCTCGGCAACGGATATCTCGG

CTCTCGCATCGATGAAGAACGTAGCGAAATGCGATACCTGGTGTGAATTGCAGAATCCCG

CGAACCATCGAGTCTTTGAACGCAAGTTGCGCCCGAGGCCTCTTGGCCGAGGGCACGCCT

GCCTGGGCGTCACGCAAAACACGCTCCCAACCCACTAACCTGGTGCGGGACGCGGCATGT

GGCTACCCGTCTCGCAAGGGGCGGTTGGCCGAAGATYCGGCTGCCGGCGTATCGTGCCGG

ACACAGCGCGTGGTAGGCGACCTCGCTTTACTAAACGCAGTGCATCCGGCGCGYAGCCAA

AGCG

>AJ240157_Festuca_glaucescens

CTGACCAAAAAAGACCGCGCACGCGTTATCCAACCTGCTGGGCGGCGGCATCGTCTGCCG

CTTGGCCAAATTCCTCGACAACCTCCCCTCTTCGGAGTTGGGGCTCGGGGTAAAAGAACC

CACGGCGCCGAAGGCGTCAAGGAACACTGTGCCTAACCCGGGGACGCGGTTGGCTTGCTG

ACCGCACCTCGAGTTGCAATGCTATATAATCCACACGACTCTCGGCAACGGATATCTCGG

CTCTCGCATCGATGAAGAACGTAGCGAAATGCGATACCTGGTGTGAATTGCAGAATCCCG

CGAACCATCGAGTCTTTGAACGCAAGTTGCGCCCGAGGCCTCTTGGCCGAGGGCACGCCT

GCCTGGGCGTCACGCAAAACACGCTCCCAACCCACTAACCTGGTGCGGGACGCGGCATGT

GGCTACCCGTCTCGCAAGGGGCGGTTGGCCGAAGATCCGGCTGCCGGCGTATCGTGCCGG

ACACAGCGCGTGGTAGGCGACCTCGCTTTACTAAACGCAGTGCATCCGGCGCGTAGCCAA

AGCG

>HM453187_Festuca_arundinacea

CTGACCAAAAAAGACCGCGCACGCGTTATCCAACCTGCTGGGCGGCGGCATCGTCTGCCG

CTTGGCCAAATTCCTCGACAACCTCCCCTCTTCGGAGTTGGGGCTCGGGGTAAAAGAACC

CACGGCGCCGAAGGCGTCAAGGAACACTGTGCCTAACCCGGGGACGCGGTTGGCTTGCTG

ACCGCACCTCGAGTTGCAATGCTATATAATCCACACGACTCTCGGCAACGGATATCTCGG

CTCTCGCATCGATGAAGAACGTAGCGAAATGCGATACCTGGTGTGAATTGCAGAATCCCG

CGAACCATCGAGTCTTTGAACGCAAGTTGCGCCCGAGGCCTCTTGGCCGAGGGCACGCCT

GCCTGGGCGTCACGCAAAACACGCTCCCAACCCACTAACCTGGTGCGGGACGCGGCATGT

GGCTACCCGTCTCGCAAGGGGCGGTTGGCCGAAGATTCGGCTGCCGGCGTATCGTGCCGG

ACACAGCGCGTGGTAGGCGACCTCGCTTTACTAAACGCAGTGCATCCGGCGCGTAGCCAA

AGCG

>HM453186_Festuca_arundinacea

CTGACCAAAAAAGACCGCGCACGCGTTATCCAACCTGCTGGGCGGCGGCATCGTCTGCCG

CTTGGCCAAATTCCTCGACAACCTCCCCTCTTCGGAGTTGGGGCTYGGGGTAAAAGAACC

CACGGCGCCGAAGGCGTCAAGGMACACTGTGCCTAACCYGGGGACGCGGTTGGCTTGCTG

ACCGCACCTCGAGTTGCAATGCTATATAATCCACACGACTCTCGGCAACGGATATCTCGG

CTCTCGCATCGATGAAGAACGTAGCGAAATGCGATACCTGGTGTGAATTGCAGAATCCCG

CGAACCATCGAGTCTTTGAACGCAAGTTGCGCCCGAGGCCTCTTGGCCGAGGGCACGCCT

GCCTGGGCGTCACGCAAAACACGCTCCCAACCCACTAACCTGGTGCGGGACGCGGCATGT

GGCTACCCGTCTCGCAAGGGGCGGTTGGCCGAAGATTCGGCTGCCGGCGTATCGTGCCGG

ACACAGCGCGTGGTAGGCGACCTCGCTTTACTAAACGCAGTGCATCCGGCGCGTAGCCAA

AGCG

>OQ346362_Festuca_pratensis_FL_34

CTGACCAAAACAGACCGCGCACGAGTTATCTAGCCCGCTGGGCGGCGGCATCGTCCGTCG

CTTGGCCAAAGTCCTCGACAACCTCATCTTTTCGGAGTTGGGGCTCGGGGTAAAAGAACC

CACGGCGCCGAAGGCGTCAAGGAACACTGTGCCTAACGAGGGGATGTGGCTGGCTTGCTG

GCCGCACCCTGAGTTGCAATTCTATATAATCCACACGACTCTCGGCAACGGATATCTCGG

CTCTCGCATCGATGAAGAACGTAGCGAAATGCGATACYTGGTGTGAATTGCAGAATCCCG

CGAACCATCGAGTCTTTGAACGCAAGTTGCGCCCGAGGCCTCTTGGCCGAGGGCACGCCT

GCCTGGGCGTCACGCCAAACACGCTCCCACCCCACTAACCTGGGGCGGGACGCGGCATGT

GGCTCCTCGTCTCGCAAGGGGCGGTGGGCCGAAGATCCGGCTGCCGGCGTATCGTGCCGG

ACACAGCGCGTGGTAGGCGACCTCGCTTTACTAAACGCAGTGCATCCGGCGCGTAGCCGA

CGCG

>OQ346363_Festuca_pratensis_FL_35

CTGACCAAAACAGACCGCGCACGAGTTATCTAGCCCGCTGGGCGGCGGCATCGTCCGTCG

CTTGGCCAAAGTCCTCGACAACCTCATCTTTTCGGAGTTGGGGCTCGGGGTAAAAGAACC

CACGGCGCCGAAGGCGTCAAGGAACACTGTGCCTAACGAGGGGATGTGGCTGGCTTGCTG

GCCGCACCCTGAGTTGCAATTCTATATAATCCACACGACTCTCGGCAACGGATATCTCGG

CTCTCGCATCGATGAAGAACGTAGCGAAATGCGATACCTGGTGTGAATTGCAGAATCCCG

CGAACCATCGAGTCTTTGAACGCAAGTTGCGCCCGAGGCCTCTTGGCCGAGGGCACGCCT

GCCTGGGCGTCACGCCAAACACGCTCCCACCCCACTAACCTGGGGCGGGACGCGGCATGT

GGCTCCTCGTCTCGCAAGGGGCGGTGGGCCGAAGATCCGGCTGCCGGCGTATCGTGCCGG

ACACAGCGCGTGGTAGGCGACCTCGCTTTACTAAACGCAGTGCATCCGGCGCGTAGCCGA

CGCG

>OQ346364_Festuca_pratensis_FL_36

CTGACCAAAACAGACCGCGCACGAGTTATCTAGCCCGCTGGGCGGCGGCATCGTCCGTCG

CTTGGCCAAAGTCCTCGACAACCTCATCTTTTCGGAGTTGGGGCTCGGGGTAAAAGAACC

CACGGCGCCGAAGGCGTCAAGGAACACTGTGCCTAACGAGGGGATGTGGCTGGCTTGCTG

GCCGCACCCTGAGTTGCAATTCTATATAATCCACACGACTCTCGGCAACGGATATCTCGG

CTCTCGCATCGATGAAGAACGTAGCGAAATGCGATACCTGGTGTGAATTGCAGAATCCCG

CGAACCATCGAGTCTTTGAACGCAAGTTGCGCCCGAGGCCTCTTGGCCGAGGGCACGCCT

GCCTGGGCGTCACGCCAAACACGCTCCCACCCCACTAACCTGGGGCGGGACGCGGCATGT

GGCTCCTCGTCTCGCAAGGGGCGGTGGGCCGAAGATCCGGCTGCCGGCGTATCGTGCCGG

ACACAGCGCGTGGTAGGCGACCTCGCTTTACTAAACGCAGTGCATCCGGCGCGTAGCCGA

CGCG

>OQ346365_Festuca_pratensis_FL_39

CTGACCAAAACAGACCGCGCACGAGTTATCTAGCCCGCTGGGCGGCGGCATCGTCCGTCG

CTTGGCCAAAGTCCTCGACAACCTCATCTTTTCGGAGTTGGGGCTCGGGGTAAAAGAACC

CACGGCGCCGAAGGCGTCAAGGAACACTGTGCCTAACGAGGGGATGTGGCTGGCTTGCTG

GCCGCACCCTGAGTTGCAATTCTATATAATCCACACGACTCTCGGCAACGGATATCTCGG

CTCTCGCATCGATGAAGAACGTAGCGAAATGCGATACCTGGTGTGAATTGCAGAATCCCG

CGAACCATCGAGTCTTTGAACGCAAGTTGCGCCCGAGGCCTCTTGGCCGAGGGCACGCCT

GCCTGGGCGTCACGCCAAACACGCTCCCACCCCACTAACCTGGGGCGGGACGCGGCATGT

GGCTCCTCGTCTCGCAAGGGGCGGTGGGCCGAAGATCCGGCTGCCGGCGTATCGTGCCGG

ACACAGCGCGTGGTAGGCGACCTCGCTTTACTAAACGCAGTGCATCCGGCGCGTAGCCGA

CGCG

>OQ346366_Festuca_pratensis_FL_40

CTGACCAAAACAGACCGCGCACGAGTTATCTAGCCCGCTGGGCGGCGGCATCGTCCGTCG

CTTGGCCAAAGTCCTCGACAACCTCATCTTTTCGGAGTTGGGGCTCGGGGTAAAAGAACC

CACGGCGCCGAAGGCGTCAAGGAACACTGTGCCTAACGAGGGGATGTGGYTGGCTTGCTG

GCCGCACCCTGAGTTGCAATTCTATATAATCCACACGACTCTCGGCAACGGATATCTCGG

CTCTCGCATCGATGAAGAACGTAGCGAAATGCGATACCTGGTGTGAATTGCAGAATCCCG

CGAACCATCGAGTCTTTGAACGCAAGTTGCGCCCGAGGCCTCTTGGCCGAGGGCACGCCT

GCCTGGGCGTCACGCCAAACACGCTCCCACCCCACTAACCTGGGGCGGGACGCGGCATGT

GGCTCCTCGTCTCGCAAGGGGCGGTGGGCCGAAGATCCGGCTGCCGGCGTATCGTGCCGG

ACACAGCGCGTGGTAGGCGACCTCGCTTTACTAAACGCAGTGCATCCGGCGCGTAGCCGA

CGCG

>AJ240151_Festuca_pratensis

CTGACCAAAACAGACCGCGCACGAGTTATCTAGCCCGCTGGGCGGCGGCATCGTCCGTCG

CTTGGCCAAAGTCCTCGACAACCTCATCTTTTCGGAGTTGGGGCTCGGGGTAAAAGAACC

CACGGCGCCGAAGGCGTCAAGGAACACTGTGCCTAACGAGGGGATGTGGCTGGCTTGCTG

GCCGCACCCTGAGTTGCAATTCTATATAATCCACACGACTCTCGGCAACGGATATCTCGG

CTCTCGCATCGATGAAGAACGTAGCGAAATGCGATACCTGGTGTGAATTGCAGAATCCCG

CGAACCATCGAGTCTTTGAACGCAAGTTGCGCCCGAGGCCTCTTGGCCGAGGGCACGCCT

GCCTGGGCGTCACGCCAAACACGCTCCCACCCCACTAACCTGGGGCGGGACGCGGCATGT

GGCTCCTCGTCTCGCAAGGGGCGGTGGGCCGAAGATCCGGCTGCCGGCGTATCGTGCCGG

ACACAGCGCGTGGTAGGCGACCTCGCTTTACTAAACGCAGTGCATCCGGCGCGTAGCCGA

CGCG

>HM453175_Festuca_pratensis

CTGACCAAAACAGACCGCGCACGAGTTATCTAGCCCGCTGGGCGGCGGCATCGTCCGTCG

CTTGGCCAAAGTCCTCGACAACCTCATCTTTTCGGAGTTGGGGCTCGGGGTAAAAGAACC

CACGGCGCCGAAGGCGTCAAGGAACACTGTGCCTAACGAGGGGATGTGGCTGGCTTGCTG

GCCGCACCCTGAGTTGCAATTCTATATAATCCACACGACTCTCGGCAACGGATATCTCGG

CTCTCGCATCGATGAAGAACGTAGCGAAATGCGATACCTGGTGTGAATTGCAGAATCCCG

CGAACCATCGAGTCTTTGAACGCAAGTTGCGCCCGAGGCCTCTTGGCCGAGGGCACGCCT

GCCTGGGCGTCACGCCAAACACGCTCCCACCCCACTAACCTGGGGCGGGACGCGGCATGT

GGCTCCTCGTCTCGCAAGGGGCGGTGGGCCGAAGATCCGGCTGCCGGCGTATCGTGCCGG

ACATAGCGCGTGGTAGGCGACCTCGCTTTACTAAACGCAGTGCATCCGGCGCGTAGCCGA

CGCG

>KF917349_Festuca_pratensis

CTGACCAAAACAGACCGCGCACGAGTTATCTAGCCCGCTGGGCGGCGGCATCGTCCGTCG

CTTGGCCAAAGTCCTCGACAACCTCATCTTTTCGGAGTTGGGGCTCGGGGTAAAAGAACC

CACGGCGCCGAAGGCGTCAAGGAACACTGTGCCTAACGAGGGGATGTGGCTGGCTTGCTG

GCCGCACCCTGAGTTGCAATTCTATATAATCCACACGACTCTCGGCAACGGATATCTCGG

CTCTCGCATCGATGAAGAACGTAGCGAAATGCGATACCTGGTGTGAATTGCAGAATCCCG

CGAACCATCGAGTCTTTGAACGCAAGTTGCGCCCGAGGCCTCTTGGCCGAGGGCACGCCT

GCCTGGGCGTCACGCCAAACACGCTCCCACCCCACTAACCTGGGGCGGGACGCGGCATGT

GGCTCCTCGTCTCGCAAGGGGCGGTGGGCCGAAGATCCGGCTGCCGGCGTATCGTGCCGG

ACACAGCGCGTGGTAGGCGACCTTGCTTTACTAAACGCAGTGTATCCGGCGCGTAGCCGA

CGCG

>KF917346_Festuca_pratensis

CTGACCAAAACAGACCGCGCACGAGTTATCTAGCCCGCTGGGCGGCGGCATCGTCCGTCG

CTTGGCCAAAGTCCTCGACAACCTCATCTTTTCGGAGTTGGGGCTCGGGGTAAAAGAACC

CACGGCGCCGAAGGCGTCAAGGAACACTGTGCCTAACGAGGGGATGTGGCTGGCTTGCTG

GCCGCACCCTGAGTTGCAATTCTATATAATCCACACGACTCTCGGCAACGGATATCTCGG

CTCTCGCATCGATGAAGAACGTAGCGAAATGCGATACCTGGTGTGAATTGCAGAATCCCG

CGAACCATCGAGTCTTTGAACGCAAGTTGCGCCCGAGGCCTCTTGGCCGAGGGCACGCCT

GCCTGGGCGTCACGCCAAACACGCTCCCACCCCACTAACCTGGGGCGGGACGCGGCATGT

GGCTCCTCGTCTCGCTAGGGGCGGTGGGCCGAAGATCCGGCTGCCGGCGTATCGTGCCGG

ACACAGCGCGTGGTAGGCGACCTCGCTTTACTAAACGCAGTGCATCCGGCGCGTAGCCGA

CGCG

>AF532948_Festuca_pratensis

CTGACCAAAACAGACCGCGCACGAGTTATCTAGCCCGCTGGGCGGCGGCATCGTCCGTCG

CTTGGCCAAAGTCCTCGACAACCTCATCTTTTCGRAGTTGGGGCTCGGGGTAAAAGAACC

CACGGCGCCGAAGGCGTCAAGGAACATTGTGCCTAACGAGGGGATGTGGCTGGCTTGCTG

GCCGCACCCTGAGTTGCAATTCTATATAATCCACACGACTCTCGGCAACGGATATCTCGG

CTCTCGCATCGATGAAGAACGTAGCGAAATGCGATACCTGGTGTGAATTGCAGAATCCCG

CGAACCATCGAGTCTTTGAACGCAAGTTGCGCCCGAGGCCTCTTGGCCGAGGGCACGCCT

GCCTGGGCGTCACGCCAAACACGCTCCCACCCCACTAACCTGGGGCGGGACGCGGCATGT

GGCTCCTCGTCTCGCAAGGGGCGGTGGGCCGAAGATCCGGCTGCCGGCGTATCGTGCCGG

ACACAGCGCGTGGTAGGCGACCTCGCTTTACTAAACGCAGTGCATCCGGCGCGTAACCGA

CGCG

>EF379046_Festuca_pratensis

CTGACCAAAACAGACCGCGCACGAGTTATCTAGCCCGCTGGGCGGCGGCATCGTCCGTCG

CTTGGCCAAAGTCCTCGACAACCTCATCTTTTCGGAGTTGGGGCTCGGGGTAAAAGAACC

CACGGCGCCGAAGGCGTCAAGGAACACTGTGCCTAACGAGGGGATGTGGCTGGCTTGCTG

GCCGCACCCTGAGTTGCAATTCTATATAGTCCACACGACTCTCGGCAACGGATATCTCGG

CTCTCGCATCGATGAAGAACGTAGCGAAATGCGATACCTGGTGTGAATTGCAGAATCCCG

CGAACCATCGAGTCTTTGAACGCAAGTTGCGCCCGAGGCCTCTTGGCCGAGGGCACGCCT

GCCTGGGCGTCACGCCAAACACGCTCCCACCCCACTAACCTGGGGCGGGATGTGGCATGT

GGCTCCTCGTCTCGCAAGGGGCGGTGGGCCGAAGATCCGGCTGCCGGCGTATCGTGCCGG

ACACAGCGCGTGGTAGGCGACCTCGCTTTACTAAACGCAGTGCATCCGGCGCGTAGCCGA

CGCG

>KF917347_Festuca_pratensis

CTGACCAAAACAGACCGCGCACGAGTTATCTAGCCCGCTGGGCGGCGGCATCGTCCGTCG

CTTGGCCAAAGTCCTCGACAACCTCATCTTTTCGGAGTTGGGGCTCGGGGTAAAAGAACC

CACGGCGCCGAAGGCGTCAAGGAACACTGTGCCTAACGAGGGGATGTGGTTGGCTTGCTG

GCCGCACCCTGAGTTGCAATTCTATATAATCCACACGACTCTCGGCAACGGATATCTCGG

CTCTCGCATCGATGAAGAACGTAGCGAAATGCGATACCTGGTGTGAATTGCAGAATCCCG

CGAACCATCGAGTCTTTGAACGCAAGTTGCGCCCGAGGCCTCTTGGCCGAGGGCACGCCT

GCCTGGGCGTCACGCCAAACACGCTCCCACCCCACTAACCTGGGGCGGGACGCGGCATGT

GGCTCCTCGTCTCGCAAGGGGCGGTGGGCCGAAGATCCGGCTGCCGGCGTATCGTGCCGG

ACACAGCGCGTGGTAGGCGACCTTGCTTTACTAAACGCAGTGCATCCGGCGCGTAGCCGA

CGCG

>KF917348_Festuca_pratensis

CTGACCAAAACAGACCGCGCACGAGTTATCTAGCCCGCTGGGCAGCGGCATCGTCCGTCG

CTTGGCCAAAGTCCTCGACAACCTCATCTTTTCGGAGTTGGGGCTCGGGGTAAAAGAACC

CACGGCGCCGAAGGCGTCAAGGAACACTGTGCCTAACGAGGGGATGTGGCTGGCTTGCTG

GCCGCACCCTGAGTTGCAATTCTATATAATCCACACGACTCTCGGCAACGGATATCTCGG

CTCTCGCATCGATGAAGAACGTAGCGAAATGCGATACCTGGTGTGAATTGCAGAATCCCG

CGAACCATCGAGTCTTTGAACGCAAGTTGCGCCCGAGGCCTCTTGGCCGAGGGCACGCCT

GCCTGGGCGTCACGCCAAACACGCTCCCACCCCACTAACCTGGGGCGGGACGCGGCATGT

GGCTCCTCGTCTCGCAAGGGGCGGTGGGCCGAAGATCCGGCTGCCGGCGTATCGTGCCGG

ACACAGCGCGTGGTAGGCGACCTCGCTTTACTAAACGCAGTGCATCCGGCGCGTAGCCGA

CGCG

>KF917345_Festuca_pratensis

CTGACCAAAACAGACCGCGCACGAGTTATCTAGCCCGCTGGGCAGCGGCATCGTCCGTCG

CTTGGCCAAAGTCCTCGACAACCTCATCTTTTCGGAGTTGGGGCTCGGGGTAAAAGAACC

CACGGCGCCGAAGGCGTCAAGGAACACTGTGCCTAACGAGGGGATGTGGCTGGCTTGCTG

GCCGCACCCTGAGTTGCAATTCTATATAATCCACACGACTCTCGGCAACGGATATCTCGG

CTCTCGCATCGATGAAGAACGTAGCGAAATGCGATACCTGGTGTGAATTGCAGAATCCCG

CGAACCATCGAGTCTTTGAACGCAAGTTGCGCCCGAGGCCTCTTGGCCGAGGGCACGCCT

GCCTGGGCGTCACGCCAAACACGCTCCCACCCCACTAACCTGGGGCGGGACGCGGCATGT

GGCTCCTCGTCTCGCTAGGGGCGGTGGGCCGAAGATCCGGCTGCCGGCGTATCGTGCCGG

ACACAGCGCGTGGTAGGCGACCTCGCTTTACTAAACGCAGTGCATCCGGCGCGTAGCCGA

CGCG

>EF379043_Festuca_pratensis

CTGATCAAAACAGACCGCGCACGAGTTATCTAGCCCGCTGGGCGGCGGCATCGTTCGTCG

CTTGGCCAAAGTCCTCGACAACCTCATCTTTTCGGAGTTGGGGCTCGGGGTAAAAGAACC

CACGGCGCCGAAGGCGTCAAGGAACACTGTGCCTAACGAGGGGATGTGGCTGGCTTGCTG

GCCGCACCCTGAGTTGCAATTCTATATAATCCACACGACTCTCGGCAACGGATATCTCGG

CTCTCGCATCGATGAAGAACGTAGCGAAATGCGATACCTGGTGTGAATTGCAGAATCCCG

CGAACCATCGAGTCTTTGAACGCAAGTTGCGCCCGAGGCCTCTTGGCCGAGGGCACGCCT

GCCTGGGCGTCACGCCAAACACGCTCCCACCCCACTAACCTGGGGCGGGACGCGGCATGT

GGCTCCTCGTCTCGCAAGGGGCGGTGGGCCGAAGATCCGGCTGCCGGCGTATCGTGCCGG

ACACAGCGCGTGGTAGGCGACCTCGCTTTACTAAACGCAGTGCATCCGGCGCGTAGCCGA

CGCG

>OQ346367_Lolium_multiflorum_FL_37

CTGACCAAAACAGACCGCGCACGAGTTATCTAGCCCGCTGGGCGGCGGCATCGTCCGTCG

CTTGGCAAAAGTCCTCGACAACCTCATCTTTTCGGAGTTGGGGCTCGGGGTAAAAGAACC

CACGGCGCCGWAGGCGTCAAGGAACACTSTGCCTAACGAGGGGATGTGGCTGGCTTGCTA

GCCGCACCCCGAGTTGCAATTCTATATAATCCACACGACTCTCGGCAACGGATATCTCGG

CTCTCGCATCGATGAAGAACGTAGCGAAATGCGATACCTGGTGTGAATTGCAGAATCCCG

CGAACCATCGAGTCTTTGAACGCAAGTTGCGCCCGAGGCCTCTTGGCCGAGGGCACGCCT

GCCTGGGCGTCACGCCAAACACGCTCCCACCCAACTAACTTGGGGTGGGACGCGGCATGT

GGCTCCTCGTCCCGCAAGGGGCGGTGGGCCAAAGATCCGGCTGCCGGCCTATCGTGCCGG

ACACAGCGCGTGGTAGGCGACCTCGCTTTACTAAACGCAGTGCCTCYGGCGCGTAGCCGA

CGCG

>OQ346368_Lolium_multiflorum_FL_42

CTGACCAAAACAGACCGCGCACGAGTTATCTAGCCCGCTGGGCGGCGGCATCGTCCGTCG

CTTGGCMAAAGTCCTCGACAACCTCATCTTTTCGGAGTTGGGGCTCGGGGTAAAAGAACC

CACGGCGCCGWAGGCGTCAAGGAACACTSTGCCTAACGAGGGGATGTGGCTGGCTTGCTR

GCCGCACCCCGAGTTGCAATTCTATATAATCCACACGACTCTCGGCAACGGATATCTCGG

CTCTCGCATCGATGAAGAACGTAGCGAAATGCGATACCTGGTGTGAATTGCAGAATCCCG

CGAACCATCGAGTCTTTGAACGCAAGTTGCGCCCGAGGCCTCTTGGCCGAGGGCACGCCT

GCCTGGGCGTCACGCCAAACACGCTCCCACCCMACTAACYTGGGGYGGGACGCGGCATGT

GGCTCCTCGTCCCGCAAGGGGCGGTGGGCCAAAGATCCGGCTGCCGGCCTATCGTGCCGG

ACACAGCGCGTGGTAGGCGACCTCGCTTTACTAAACGCAGTGCCTCCGGCGCGTWRCCGA

CGCG

>OQ346369_Lolium_multiflorum_FL_43

CTGACCAAAACAGACCGCGCACGAGTTATCTAGCCCGCTGGGCGGCGGCATCGTCCGTCG

CTTGGCMAAAGTCCTCGACAACCTCATCTTTTCGGAGTTGGGGCTCGGGGTAAAAGAACC

CACGGCGCCGWAGGCGTCAAGGAACACTSTGCCTAACGAGGGGATGTGGCTGGCTTGCTR

GCCGCACCCCGAGTTGCAATTCTATATAATCCACACGACTCTCGGCAACGGATATCTCGG

CTCTCGCATCGATGAAGAACGTAGCGAAATGCGATACCTGGTGTGAATTGCAGAATCCCG

CGAACCATCGAGTCTTTGAACGCAAGTTGCGCCCGAGGCCTCTTGGCCGAGGGCACGCCT

GCCTGGGCGTCACGCCAAACACGCTCCCACCCMACTAACYTGGGGYGGGACGCGGCATGT

GGCTCCTCGTCCCGCAAGGGGCGGTGGGCCAAAGATCCGGCTGCCGGCCTATCGTGCCGG

ACACAGCGCGTGGTAGGCGACCTCGCTTTACTAAACGCAGTGCCTCCGGYGCGTAGCCGA

CGCG

>KJ598998_Lolium_multiflorum

CTGACCAAAACAGACCGCGCACGAGTTATCTAGCC-GCTGGGCGGCGGCATCGTCCGTCG

CTTGGCAAAAGTCCTCGACAACCTCATCTTTTCGGAGTTGGGGCTCGGGGTAAAAGAACC

CACGGCGCCGAAGGCGTCAAGGAACACTGTGCCTAACGAGGGGATGTGGCTGGCTTGCTA

GCCGCACCCCGAGTTGCAATTCTATATAATCCACACGACTCTCGGCAACGGATATCTCGG

CTCTCGCATCGATGAAGAACGTAGCGAAATGCGATACCTGGTGTGAATTGCAGAATCCCG

CGAACCATCGAGTCTTTGAACGCAAGTTGCGCCCGAGGCCTCTTGGCCGAGGGCACGCCT

GCCTGGGCGTCACGCCAAACACGCTCCCACCCAACTAACTTGGGGTGGGACGCGGCATGT

GGCTCCTCGTCCCGCAAGGGGCGGTGGGCCAAAGATCCGGCTGCCGGCCTATCGTGCCGG

ACACAGCGCGTGGTAGGCGACCTCGCTTTACTAAACGCAGTGCCTCCGGCGCGTAGCCGA

CGCG

>AB126353_Lolium_multiflorum

CTGACCAAAACAGACCGCGCACGAGTTATCTAGCCNGCTGGGCGGCGGCATCGTCNGTCG

CTTGGCAAAAGTCCTCGACAACCTCATCTTTTCGGAGTTGGGGCTCGGGGTAAAAGAACC

CACGGCGCCGAAGGCGTCAAGGAACACTGTGCCTAACGAGGGGATGTGGCTGGCTTGCTA

GCCGCACCCCGAGTTGCAATTCTATATAATCCACACGACTCTCGGCAACGGATATCTCGG

CTCTCGCATCGATGAAGAACGTAGCGAAATGCGATACCTGGTGTGAATTGCAGAATCCCG

CGAACCATCGAGTCTTTGAACGCAAGTTGCGCCCGAGGCCTCTTGGCCGAGGGCACGCCT

GCCTGGGCGTCACGCCAAACACGCTCCCACCCAACTAACTTGGGGTGGGACGCGGCATGT

GGCTCCTCGTCCCGCAAGGGGCGGTGGGCCAAAGATCCGGCTGCCGGCCTATCGTGCCGG

ACACAGCGCGTGGTAGGCGACCTCGCTTTACTAAACGCAGTGCCTCCGGCGCGTAGCCGA

CGCG

>AJ240140_Lolium_multiflorum

CTGACCAAAACAGACCGCGCACGAGTTATCTAGCCCGCTGGGCGGCGGCATCGTCCGTCG

CTTGGCAAAAGTCCTCGACAACCTCATCTTTTCGGAGTTGGGGCTCGGGGTAAAAGAACC

CACGGCGCCGTAGGCGTCAAGGAACACTGTGCCTAACGAGGGGATGTGGCTGGCTTGCTA

GCCGCACCCCGAGTTGCAATTCTATATAATCCACACGACTCTCGGCAACGGATATCTCGG

CTCTCGCATCGATGAAGAACGTAGCGAAATGCGATACCTGGTGTGAATTGCAGAATCCCG

CGAACCATCGAGTCTTTGAACGCAAGTTGCGCCCGAGGCCTCTTGGCCGAGGGCACGCCT

GCCTGGGCGTCACGCCAAACACGCTCCCACCCAACTAACTTGGGGTGGGACGCGGCATGT

GGCTCCTCGTCCCGCAAGGGGCGGTGGGCCAAAGATCCGGCTGCCGGCCTATCGTGCCGG

ACACAGCGCGTGGTAGGCGACCTCGCTTTACTAAACGCAGTGCCTCCGGCGCGTAGCCGA

CGCG

>AF532946_Lolium_multiflorum

CTGACCAAAACAGACCGCGCACGAGTTATCTAGCCCGCTGGGCGGCGGCATCGTCCGTCG

CTTGGCAAAAGTCCTCGACAACCTCATCTTTTCGGAGTTGGGGCTCGGGGTAAAAGAACC

CACGGCGCCGTAGGCGTCAAGGAACACTGTGCCTAACGAGGGGATGTGGCTGGCTTGCTA

GCCGCACCCCGAGTTGCAATTCTATATAATCCACACGACTCTCGGCAACGGATATCTCGG

CTCTCGCATCGATGAAGAACGTAGCGAAATGCGATACCTGGTGTGAATTGCAGAATCCCG

CGAACCATCGAGTCTTTGAACGCAAGTTGCGCCCGAGGCCTCTTGGCCGAGGGCACGCCT

GCCTGGGCGTCACGCCAAACACGCTCCCACCCAACTAACTTGGGGTGGGACGCGGCATGT

GGCTCCTCGTCCCGCAAGGGGCGGTGGGCCAAAGATCCGGCTGCCGGCCTATCGTGCCGG

ACACAGCGCGTGGTAGGCGACCTCGCTTTACTAAACGCAGTGCCTCCGGCGCGTARCCGA

CGCG

>EF379072_Lolium_multiflorum

CTGACCAAAACAGACCGCGCACGAGTTATCTAGCCCGCTGGGCGGCGGCATCGTCCGTCG

CTTGGCAAAAGTCCTCGACAACCTCATCTTTTCGGAGTTGGGGCTCGGGGTAAAAGAACC

CACGGCGCCGTAGGCGTCAAGGAACACTGTGCCTAACGAGGGGATGTGGCTGGCTTGCTA

GCCGCACCCCGAGTTGCAATTCTATATAATCCACACGACTCTCGGCAACGGATATCTCGG

CTCTCGCATCGATGAAGAACGTAGCGAAATGCGATACCTGGTGTGAATTGCAGAATCCCG

CGAACCATCGAGTCTTTGAACGCAAGTTGCGCCCGAGGCCTCTTGGCCGAGGGCACGCCT

GCCTGGGCGTCACGCCAAACACGCTCCCACCCAACTAACTTGGGGTGGGACGCGGCATGT

GGCTCCTCGTCCCGCAAGGGGCGGTGGGCCAAAGATCCGGCTGC-GGCCTATCGTGCCGG

ACACAGCGCGTGGTAGGCGACCTCGCTTTACTAAACGCAGTGCCTCCGGCGCGTAGCCGA

CGCG

>HM453190_Lolium_multiflorum

CTGACCAAAACAGACCGCGCACGAGTTATCTAGCCCGCTGGGCGGCGGCATCGTCCGTCG

CTTGGCAAAAGTCCTCGACAACCTCATCTTTTCGGAGTTGGGGCTCGGGGTAAAAGAACC

CACGGCGCCGWAGGCGTCAAGGAACACTGTGCCTAACGAGGGGATGTGGCTGGCTTGCTA

GCCGCACCCCGAGTTGCAATTCTATATAATCCACACGACTCTCGGCAACGGATATCTCGG

CTCTCGCATCGATGAAGAACGTAGCGAAATGCGATACCTGGTGTGAATTGCAGAATCCCG

CGAACCATCGAGTCTTTGAACGCAAGTTGCGCCCGAGGCCTCTTGGCCGAGGGCACGCCT

GCCTGGGCGTCACGCCAAACACGCTCCCACCCAACTAACTTGGGGTGGGACGCGGCATGT

GGCTCCTCGTCCCGCAAGGGGCGGTGGGCCAAAGATCCGGCTGCCGGCCTATCGTGCCGG

ACACAGCGCGTGGTAGGCGACCTCGCTTTACTAAACGCAGTGCCTCCGGCGCGTAGCCGA

CGCG

>EF379070_Lolium_multiflorum

CTGACCAAAACAGACCGCGCACGAGTTATCTAGCCCGCTGGGCGGCGGCATCGTCCGTCG

CTTGGCAAAAGTCCTCGACAACCTCATCTTTTCGGAGTTGGGGCTCGGGGTAAAAGAACC

CACGGCGCCGAAGGCGTCAAGGAACACTGTGCCTAACGAGGGGATGTGGCTGGCTTGCTA

GCCGCACCCCGAGTTGCAATTCTATATAATCCACACGACTCTCGGCAACGGATATCTCGG

CTCTCGCATCGATGAAGAACGTAGCGAAATGCGATACCTGGTGTGAATTGCAGAATCCCG

CGAACCATCGAGTCTTTGAACGCAAGTTGCGCCCGAGGCCTCTTGGCCGAGGGCACGCCT

GCCTGGGCGTCACGCCAAACACGCTCCCACCCAACTAACTTGGGGTGGGACGCGGCATGT

GGCTCCTCGTCCCGCAAGGGGCGGTGGGCCAAAGATCCGGCTGCCGGCCTATCGTGCCGG

AAACAGCGCGTGGTAGGCGACCTCGCTTTACTAAACGCAGTGCCTCCGGCGCGTAGCCGA

CGCG

>AJ240139_Lolium_multiflorum

CTGACCAAAACAGACCGCGCACGAGTTATCTAGCCCGCTGGGCGGCGGCATCGTCCGTCG

CTTGGCAAAAGTCCTCGACAACCTCATCTTTTCGGAGTTGGGGCTCGGGGTAAAAGAACC

CACGGCGCCGAAGGCGTCAAGGAACACTGTGCCTAACGAGGGGATGTGGCTGGCTTGCTA

GCCGCACCCCGAGTTGCAATTCTATATAATCCACACGACTCTCGGCAACGGATATCTCGG

CTCTCGCATCGATGAAGAACGTAGCGAAATGCGATACCTGGTGTGAATTGCAGAATCCCG

CGAACCATCGAGTCTTTGAACGCAAGTTGCGCCCGAGGCCTCTTGGCCGAGGGCACGCCT

GCCTGGGCGTCACGCCAAACACGCTCCCACCCAACTAACTTGGGGTGGGACGCGGCATGT

GGCTCCTCGTCCCGCAAGGGGCGGTGGGCCAAAGATCCGGCTGCCGGCCTATCGTGCCGG

ACACAGCGCGTGGTAGGCGACCTCGCTTTACTAAACGCAGTGCCTCCGGCGCGTAGCCGA

CGCG

>OQ346370_Lolium_perenne_FL_38

CTGACCAAAACAGACCGCGCACGAGTTATCTAGCCCGCTGGGCGGCGGCATCGTCCGTCG

CTTGGCAAAAGTCCTCGACAACCTCATCTTTTCGGAGTTGGGGCTCGGGGTAAAAGAACC

CACGGCGCCGAAGGCGTCAAGGAACACTGTGCCTAACGAGGGGATGTGGCTGGCTTGCTA

GCCGCACCCCGAGTTGCAATTCTATATAATCCACACGACTCTCGGCAACGGATATCTCGG

CTCTCGCATCGATGAAGAACGTAGCGAAATGCGATACCTGGTGTGAATTGCAGAATCCCG

CGAACCATCGAGTCTTTGAACGCAAGTTGCGCCCGAGGCCTCTTGGCCGAGGGCACGCCT

GCCTGGGCGTCACGCCAAACACGCTCCCACCCAACTAACTTGGKGTGGGACGCGGCATGT

GGCTCCTCRTCCCGCAAGGGGCGGTGGGCCAAAGATCCGGCTGCCGGCCTATCGTGCCGG

ACACAGCGCGTGGTAGGCGACCTCGCTTTACTAAACGCAGTGCCTCTGGCGCGTAGCCGA

CGCG

>KJ598999_Lolium_perenne

CTGACCAAAACAGACCGCGCACGAGTTATCTAGCCTGCTGGGCGGCGGCATCGTCTGTCG

CTTGGCAAAAGTCCTCGACAACCTCATCTTTTCGGAGTTGGGGCTCGGGGTAAAAGAACC

CACGGCGCCGAAGGCGTCAAGGAACACTGTGCCTAACGAGGGGATGTGGCTGGCTTGCTA

GCCGCACCCCGAGTTGCAATTCTATATAATCCACACGACTCTCGGCAACGGATATCTCGG

CTCTCGCATCGATGAAGAACGTAGCGAAATGCGATACCTGGTGTGAATTGCAGAATCCCG

CGAACCATCGAGTCTTTGAACGCAAGTTGCGCCCGAGGCCTCTTGGCCGAGGGCACGCCT

GCCTGGGCGTCACGCCAAACACGCTCCCACCCAACTAACTTGGGGTGGGACGCGGCATGT

GGCTCCTCGTCCCGCAAGGGGCGGTGGGCCAAAGATCCGGCTGCCGGCCTATCGTGCCGG

ACACAGCGCGTGGTAGGCGACCTCGCTTTACTAAACGCAGTGCCTCCGGCGCGTAGCCGA

CGCG

>KF454873_Lolium_perenne

CTGACCAAAACAGACCGCGCACGAGTTATCTAGCCCGCTGGGCGGCGGCATCGTCCGTCG

CTTGGCAAAAGTCCTCGACAACCTCATCTTTTCGGAGTTGGGGCTCGGGGTAAAAGAACC

CACGGCGCCGTAGGCGTCAAGGAACACTGTGCCTAACGAGGGGATGTGGCTGGCTTGCTA

GCCGCACCCCGAGTTGCAATTCTATATAATCCACACGACTCTCGGCAACGGATATCTCGG

CTCTCGCATCGATGAAGAACGTAGCGAAATGCGATACCTGGTGTGAATTGCAGAATCCCG

CGAACCATCGAGTCTTTGAACGCAAGTTGCGCCCGAGGCCTCTTGGCCGAGGGCACGCCT

GCCTGGGCGTCACGCCAAACACGCTCCCACCCAACTAACTTGGGGTGGGACGCGGCATGT

GGCTCCTCGTCCCGCAAGGGGCGGTGGGCCAAAGATCCGGCTGCCGGCCTATCGTACCGG

ACACAGCGCGTGGTAGGCGACCTCGCTTTACTAAACGCAGTGCCTCTGGCGCGTAGCCGA

CGCG

>KU883513_Lolium_perenne

CTGACCAAAACAGACCGCGCACGAGTTATCTAGCCCGCTGGGCGGCGGCATCGTCCGTCG

CTTGGCAAAAGTCCTCGACAACCTCATCTTTTCGGAGTTGGGGCTCGGGGTAAAAGAACC

CACGGCGCCGTAGGCGTCAAGGAACACTGTGCCTAACGAGGGGATGTGGCTGGCTTGCTA

GCCGCACCCCGAGTTGCAATTCTATATAATCCACACGACTCTCGGCAACGGATATCTCGG

CTCTCGCATCGATGAAGAACGTAGCGAAATGCGATACCTGGTGTGAATTGCAGAATCCCG

CGAACCATCGAGTCTTTGAACGCAAGTTGCGCCCGAGGCCTCTTGGCCGAGGGCACGCCT

GCCTGGGCGTCACGCCAAACACGCTCCCACCCAACTAACTTGGGGTGGGACGCGGCATGT

GGCTCCTCGTCCCGCAAGGGGCGGTGGGCCAAAGATCCGGCTGCCGGCCTATCGTGCCGG

ACACAGCGCGTGGTAGGCGACCTCGCTTTACTAAACGCAGTGCCTCCGGCGCGTAGCCGA

CGCG

>EF379075_Lolium_perenne

CTGACCAAAACAGACCGCGCACGAGTTATCTAGCCCGCTGGGCGGCGGCATCGTCCGTCG

CTTGGCAAAAGTCCTCGACAACCTCATCTTTTCGGAGTTGGGGCTCGGGGTAAAAGAACC

CACGGCGCCGAAGGCGTCAAGGAACACTGTGCCTAACGAGGGGATGTGGCTGGCTTGCTA

GCCGCACCCCGAGTTGCAATTCTATATAATCCACACGACTCTCGGCAACGGATATCTCGG

CTCTCGCATCGATGAAGAACGTAGCGAAATGCGATACCTGGTGTGAATTGCAGAATCCCG

CGAACCATCGAGTCTTTGAACGCAAGTTGCGCCCGAGGCCTCTTGGCCGAGGGCACGCCT

GCCTGGGCGTCACGCCAAACACGCTCCCACCCAACTAACTTGGGGTGGGACGCGGCATGT

GGCTCCTCGTCCCGCAAGGGGCGGTGGGCCAAAGATCCGGCTGCCGGCCTATCGTGCCGG

ACACAGCGCGTGGTAGGCGACCTCGGTTTACTAAACGCAGTGCCTCTGGCGCGTAGCCGA

CCCG

>EF379073_Lolium_perenne

CTGACCAAAACAGACCGCGCACGAGTTATCTAGCCCGCTGGGCGGCGGCATCGTCCGTCG

CTTGGCAAAAGTCCTCGACAACCTCATCTTTTCGGAGTTGGGGCTCGGGGTAAAAGAACC

CACGGCGCCGAAGGCGTCAAGGAACACTGTGCCTAACGAGGGGATGTGGCTGGCTTGCTA

GCCGCACCCCGAGTTGCAATTCTATATAATCCACACGACTCTCGGCAACGGATATCTCGG

CTCTCGCATCGATGAAGAACGTAGCGAAATGCGATACCTGGTGTGAATTGCAGAATCCCG

CGAACCATCGAGTCTTTGAACGCAAGTTGCGCCCGAGGCCTCTTGGCCGAGGGCACGCCT

GCCTGGGCGTCACGCCAAACACGCTCCCACCCAACTAACTTGGGGTGGGACGCGGCATGT

GGCTCCTCGTCCCGCAAGGGGCGGTGGGCCAAAGATCCGGCTGCCGGCCTATCGTGCCGG

ACACAGCGCGTGGTAGGCGACCTCGCTTTACTAAACGCAGTGCCTCTGGCGCGTAGCCGA

CGCG
